# Petroselinic Acid from Apiaceae Family Plants Ameliorates Autoimmune Disorders Through Suppressing Cytosolic-Nucleic-Acid-Mediated Type I Interferon Signaling

**DOI:** 10.3390/biom15030329

**Published:** 2025-02-24

**Authors:** Yue Guo, Yun-Ying Wang, Yao Wang, Yan-Hong Liu, Jia-Yu Liu, Yan-Yan Shen, Ai-Ping Cao, Rui-Bo Wang, Bo-Yang Xie, Xin Pan, Ai-Ling Li, Tao Zhou, Na Wang, Qing Xia, Wei-Na Zhang

**Affiliations:** Nanhu Laboratory, National Center of Biomedical Analysis, Beijing 100039, China; guoyue8899@163.com (Y.G.); 3170103695@zju.edu.cn (Y.-Y.W.); ywang@xmail.ncba.ac.cn (Y.W.); lyhyjyy@163.com (Y.-H.L.); liujiayu6066@163.com (J.-Y.L.); 18997269090@163.com (Y.-Y.S.); 12118135@zju.edu.cn (A.-P.C.); 18810622983@163.com (R.-B.W.); xby19990318@163.com (B.-Y.X.); xpan@ncba.ac.cn (X.P.); alli@ncba.ac.cn (A.-L.L.); tzhou@ncba.ac.cn (T.Z.); nwang@ncba.ac.cn (N.W.)

**Keywords:** petroselinic acid, Apiaceae family plants, type I interferon, autoimmune disease

## Abstract

The recognition of cytosolic nucleic acids is a critical step in the host immune response against danger signals, such as molecular patterns from pathogens or tissue damage. Nonetheless, over-reactivity to self-nucleic acids leads to the sustained production of type I interferon (IFN), mediated either by cGAS or RLR, contributing to the pathogenesis of certain autoimmune diseases, such as Aicardi–Goutières syndrome (AGS). Therefore, inhibiting excessive IFN production represents a potential therapeutic strategy for such autoimmune conditions. In this study, we discovered that petroselinic acid (PA), a natural compound isolated from Apiaceae family plants, effectively suppresses type I IFN production induced by cytosolic nucleic acids. Mechanistic investigations revealed that PA inhibits the phosphorylation of TBK1 and IRF3, which are key nodal proteins within the type I interferon pathway. Notably, molecular docking suggests potential binding between PA and cytosolic nucleic acid sensors, such as cGAS and RIG-I. Moreover, we found that PA effectively attenuates the expression of type I IFN and their downstream interferon-stimulated genes (ISGs) in models of AGS autoimmune disease characterized by excessive nucleic acid accumulation. Thus, our research identifies a natural compound that offers a promising strategy for treating autoimmune diseases resulting from aberrant self-nucleic acid recognition and the hyperactivation of type I interferon.

## 1. Introduction

The innate immune system serves as the primary defense mechanism against pathogens, with cytosolic nucleic acid recognition playing a central role in immune responses. Pattern recognition receptors (PRRs), such as RIG-I, MDA5, and cGAS, detect cytosolic RNA and DNA, respectively, and initiate signaling pathways leading to the production of type I interferon (IFN), including IFN-α and IFN-β [1,2]. Type I IFNs activate immune cells and induce interferon-stimulated gene (ISG) expression to respond to danger signals [2]. However, the excessive accumulation of endogenous nucleic acids under certain conditions could trigger an abnormally high production of type I interferon, contributing to chronic inflammation and autoimmune diseases like Aicardi–Goutières syndrome and systemic lupus erythematosus [3,4,5,6].

Upon sensing cytosolic dsRNA, RIG-I like receptors (RLRs) undergo conformational changes to recruit a signaling adaptor, named mitochondrial antiviral-signaling (MAVS) protein, which initiates MAVS-TBK1-IRF3 cascade activation [7]. While RIG-I primarily recognizes short dsRNA and 5′-triphosphate RNA, MDA5 is specialized in detecting long dsRNA structures [8,9,10]. On the other hand, cGAS detects cytosolic DNA and catalyzes the production of cyclic GMP-AMP (cGAMP), which activates the STING-TBK1-IRF3 pathway [11]. These pathways are vital for host defense against pathogens. However, their dysregulation can exacerbate inflammation and contribute to autoimmune disease progression. Therefore, understanding how to regulate these pathways and suppress excessive IFN responses is crucial for developing effective treatments for autoimmune diseases.

Recent studies targeting cytosolic nucleic-acid-induced type I interferon production have shown promising potential for controlling autoimmune diseases. Genetic approaches, such as CRISPR, have demonstrated significant efficacy in correcting gene mutations or dysregulation within key modulators of cytosolic nucleic acid sensing pathways, thereby reducing aberrant IFN production and attenuating symptoms of autoimmune disorders [12]. Small-molecule drugs like aspirin have exhibited notable effects on Aicardi–Goutières syndrome (AGS) by inhibiting cGAS activity and reducing interferon signaling [13]. Importantly, natural products, such as epigallocatechin gallate (EGCG) from green tea and curcumin from turmeric, have also been found to modulate these sensing pathways, affecting interferon-associated immune dysregulation [14,15]. EGCG inhibits the binding between G3BP1 and cGAS, suppressing cGAS activation, while curcumin attenuates IRF3 activation. These findings highlight the therapeutic potential of natural compounds for interferon-related immune diseases.

Petroselinic acid, a monounsaturated fatty acid isolated from the seeds of Apiaceae family plants, has shown potential health benefits in various applications. Extracts containing petroselinic acid are noted for their antioxidant properties, and related fatty acids within the same family have been associated with therapeutic advantages for cardiovascular health [16,17]. Furthermore, petroselinic acid itself has demonstrated specific benefits in managing metabolic conditions, such as diabetes [18]. Despite its established biological activities, its involvement in modulating innate immune signaling pathways, especially those linked to type I interferon responses, remains poorly understood.

In this study, through employing a screening approach based on the IFN-stimulated response element (ISRE) luciferase reporter system, we identified the natural compound petroselinic acid as a novel regulator for the cytosolic nucleic acid sensing pathway. We observed that petroselinic acid effectively inhibits signal activation induced by cytosolic nucleic acids. Importantly, petroselinic acid efficiently attenuates type I interferon and ISGs production in various AGS models. These findings not only unveil an unreported function of petroselinic acid but also provide a new strategy for the treatment of autoimmune diseases associated with dysregulated interferon signaling.

## 2. Materials and Methods

### 2.1. Antibodies and Reagents

Anti-phospho-TBK1 (5483S), anti-phospho-IRF-3 (37829S), anti-TBK1 (3504S), and anti-ADAR1 (D7E2M) were obtained from Cell Signaling Technology, Inc. (Danvers, MA, USA). Anti-IRF3 (ab76493) was obtained from Abcam plc (Cambridge, UK). Anti-Trex1 (611986) was obtained from BD Biosciences (San Jose, CA, USA). The dilution ratio of the above-mentioned antibodies was 1:1000. Anti-GAPDH (10494-1-AP) and anti-Tubulin (11224-1-AP) were from Proteintech Group, Inc. (Rosemont, IL, USA). The dilution ratio of the above-mentioned antibodies was 1:5000. Anti-β-Actin (sc-47778) was obtained from Santa Cruz Biotechnology, Inc. (Dallas, TX, USA), and the dilution ratio of it was 1:200.

Petroselinic acid (HY-113362), procured from MedChemExpress (Monmouth Junction, NJ, USA), was dissolved in DMSO to prepare a stock solution with a concentration of 300 mM, and further diluted into different doses as indicated. Recombinant M-CSF (416-ML) was obtained from R&D Systems, Inc. (Minneapolis, MN, USA). Lipofectamine 2000 Transfection Reagent (31985070) and Lipofectamine RNAiMAX Transfection Reagent (13778100) were obtained from Invitrogen (Carlsbad, CA, USA). Poly(I:C) (tlrl-picw) was obtained from InvivoGen (San Diego, CA, USA). Lipopolysaccharide (LPS, 437628), HT-DNA (D6898), and TRI reagent (93289) were obtained from Sigma-Aldrich (St. Louis, MO, USA). Petroselinic acid (HY-113362) was obtained from MedChemExpress. PrimeScript RT Master Mix (RR036A) was obtained from Takara Bio Inc. (Kusatsu, Shiga, Japan). PowerUp SYBR Green Master Mix (A25778) was obtained from Applied Biosystems (Carlsbad, CA, USA).

### 2.2. Mice

All animal experiments in this study were conducted in compliance with relevant ethical regulations and were approved by the Institutional Animal Care and Use Committee (IACUC) of the National Center of Biomedical Analysis. C57BL/6 mice (stock: 219) were purchased from Beijing Vital River Laboratory Animal Technology (Beijing, China), and Trex1^−/−^ C57BL/6 mice were from D. Barnes and T. Lindahl (Cancer Research UK, London, UK) [19,20]. Mice were housed under specific pathogen-free (SPF) conditions at 21 ± 1 °C and 60 ± 5% humidity, with a 12 h light/dark cycle. Experimental and control groups were housed separately. Mice aged 6–10 weeks were used for experiments and euthanized using carbon dioxide in accordance with ethical guidelines.

### 2.3. Cell Culture

A549, U937, and BJ5ta cells were obtained from the American Type Culture Collection (ATCC, Manassas, VA, USA). A549 and U937 cells were cultured in RPMI-1640 medium supplemented with 10% fetal bovine serum, 1% penicillin-streptomycin, and maintained at 37 °C in a humidified incubator with 5% CO_2_. BJ5ta cells were cultured in Dulbecco’s Modified Eagle Medium (DMEM) supplemented with 10% FBS and 1% penicillin-streptomycin at 37 °C under similar conditions. All cell lines were passaged every 2–3 days to maintain optimal growth conditions.

### 2.4. Differentiation of Bone-Marrow-Derived Macrophages (BMDMs)

Bone marrow was harvested from the femurs of 6-to-8-week-old C57BL/6J mice. The bone marrow cells were directly plated in 15 cm dishes at a density of 1 × 10^6^ cells per mL in DMEM supplemented with 10% FBS, 1% penicillin-streptomycin, and 10 ng/mL recombinant M-CSF (R&D Systems) to induce macrophage differentiation. Cells were incubated at 37 °C in a humidified 5% CO_2_ incubator. A 5 mL medium with M-CSF was supplemented on the third day, and fully differentiated macrophages were harvested for experiments on days 5–6.

### 2.5. Cell Transfection and Stimulation

A549 (2 × 10^5^ cells/well of 12-well plate), U937 (1 × 10^6^ cells/well of 12-well plate), BJ5ta (2 × 10^5^ cells/well of 12-well plate), and BMDM (1 × 10^6^ cells/well of 12-well plate) cells were transfected with Poly(I:C) or HT-DNA using Lipofectamine 2000 Transfection Reagent (Invitrogen) at a final concentration of 1 μg/mL for 6 h, according to the manufacturer’s protocol. U937 and BMDM cells were stimulated with 1 μg/mL LPS (Sigma-Aldrich, St. Louis, MO, USA) for 4 h to induce an inflammatory response. BJ5ta cells were stimulated with cGAMP as follows: cells were incubated with 2 μg/mL cGAMP in permeabilization buffer (50 mM HEPES, pH 7.0; 100 mM KCl; 3 mM MgCl_2_; 0.1 mM DTT; 85 mM sucrose; 0.2% BSA; 1 mM ATP; and 0.1 mM GTP) supplemented with 1 mg/mL digitonin (Sigma, D141) for 30 min at 37 °C. Following incubation, the permeabilization buffer was replaced with DMEM, and cells were further cultured for another 3 h before harvest.

### 2.6. RNA Isolation and Quantitative PCR (qPCR)

Total RNA was isolated from cells using TRI reagent (Sigma-Aldrich). A total of 500 ng of RNA was reverse transcribed into cDNA using the PrimeScript RT Master Mix (TaKaRa). qPCR was performed using PowerUp SYBR Green Master Mix (Applied Biosystems) on a StepOnePlus Real-Time PCR System (Applied Biosystems) according to the manufacturer’s instructions. Data were analyzed using StepOnePlus v2.2 software. GAPDH was used as an internal control for normalization. The primers used were as follows:

mIfnb-Fwd: 5′-TCCGAGCAGAGATCTTCAGGAA-3′

mIfnb-Rev: 5′-TGCAACCACCACTCATTCTGAG-3′

mCxcl10-Fwd: 5′-GCCGTCATTTTCTGCCTCA-3′

mCxcl10-Rev: 5′-CGTCCTTGCGAGAGGGATC-3′

mIsg15-Fwd: 5′-TGACTGTGAGAGCAAGCAGC-3′

mIsg15-Rev: 5′-CCCCAGCATCTTCACCTTTA-3′

mIfit1-Fwd: 5′-GAACCCATTGGGGATGCACAACCT-3′

mIfit1-Rev: 5′-CTTGTCCAGGTAGATCTGGGCTTCT-3′

mIfit2-Fwd: 5′-ATGAGTTTCAGAACAGTGAGTTTAA-3′

mIfit2-Rev: 5′-AACTGGCCCATGTGATAGTAGACCC-3′

mRsad2-Fwd: 5′-CCCGTGAGTGTCAACTACCAC-3′

mRsad2-Rev: 5′-GCCCAAGTATTCACCCCTGTC-3′

mUsp18-Fwd: 5′-TTGGGCTCCTGAGGAAACC-3′

mUsp18-Rev: 5′-CGATGTTGTGTAAACCAACCAGA-3′

m Oas1a-Fwd: 5′-GCCTGATCCCAGAATCTATGC-3′

m Oas1a-Rev: 5′-GAGCAACTCTAGGGCGTACTG-3′

mActin-Fwd: 5′-CATTGCTGACAGGATGCAGAAGG -3′

mActin-Rev: 5′-TGCTGGAAGGTGGACAGTGAGG-3′

mHprt-Fwd: 5′-CAGTCCCAGCGTCGTGATTAG-3′

mHprt-Rev: 5′-AAACACTTTTTCCAAATCCTCGG-3′

hIFNB-Fwd: 5′-GCTTGGATTCCTACAAAGAAGCA-3′

hIFNB-Rev: 5′-ATAGATGGTCAATGCGGCGTC-3′

hCXCL10-Fwd: 5′-GTGGCATTCAAGGAGTACCTC-3′

hCXCL10-Rev: 5′-TGATGGCCTTCGATTCTGGATT-3′

hISG15-Fwd: 5′-CGCAGATCACCCAGAAGATCG-3′

hISG15-Rev: 5′-TTCGTCGCATTTGTCCACCA-3′

hIFIT1-Fwd: 5′-GCGCTGGGTATGCGATCTC-3′

hIFIT1-Rev: 5′-CAGCCTGCCTTAGGGGAAG-3′

hIFIT2-Fwd: 5′-AAGCACCTCAAAGGGCAAAAC-3′

hIFIT2-Rev: 5′-TCGGCCCATGTGATAGTAGAC-3′

hRSAD2-Fwd: 5′-TGGGTGCTTACACCTGCTG-3′

hRSAD2-Rev: 5′-GAAGTGATAGTTGACGCTGGTT-3′

hUSP18-Fwd: 5′-CCTGAGGCAAATCTGTCAGTC-3′

hUSP18-Rev: 5′-CGAACACCTGAATCAAGGAGTTA-3′

hGAPDH-Fwd: 5′-GGAGCGAGATCCCTCCAAAAT-3′

hGAPDH-Rev: 5′-GGCTGTTGTCATACTTCTCATGG-3′

### 2.7. Immunoblot Analysis

Cells were lysed in lysis buffer (20 mM Tris-HCl, pH 7.5, 0.5% NP40, 250 mM NaCl, 3mM EDTA, and 3 mM EGTA) supplemented with a protease inhibitor cocktail. The lysates were centrifuged at 15,000 rpm for 15 min at 4 °C, and the supernatants were collected. Protein samples were mixed with 5× SDS-loading buffer and separated by electrophoresis on a 10% polyacrylamide gel. The proteins were then transferred to PVDF membranes. The membranes were blocked with 5% skim milk and incubated with the appropriate primary antibodies. Protein signals were visualized using chemiluminescence detection.

### 2.8. RNA Interference

siRNA-mediated knockdown in BJ5ta cells (1 × 10^5^ cells/well of 12-well plate) was performed using Lipofectamine RNA iMAX Transfection Reagent (Invitrogen) according to the manufacturer’s instructions. siRNAs were transfected at a final concentration of 100 nM. After 24 h of transfection, cells were treated with PA for an additional 24 h. The drug-containing medium was replaced every 12 h. Human ADAR1-specific siRNA (5′-GCAGAGTCAGCATATATGA-3′) and control siRNA were synthesized by Sangon Biotech (Shanghai, China).

### 2.9. Screening of the Small-Molecule Library

A lentiviral reporter system was constructed to monitor ISRE activation by modifying the pGreenFire1-ISRE Lentivector plasmid (System Biosciences, Palo Alto, CA, USA) to express GFP under the control of the ISRE. Lentiviral particles were produced by co-transfecting the modified plasmid with packaging plasmids into HEK293T cells for 48 h. Subsequently, A549 cells were infected with the lentivirus for 24 h and selected using 800 μg/mL G418 (Thermo Fisher Scientific, Waltham, MA, USA) to generate a stable ISRE-GFP reporter cell line. For the screening assay, the stable A549-ISRE-GFP cells were plated at a density of 20,000 cells per well in 96-well plates. Cells were pretreated with small individual molecules and then stimulated with poly(I:C) (1 μg/mL) to activate the ISRE pathway. After 15 min of nuclear staining with DAPI, images were captured using the Opera Phenix High-Content Screening System (PerkinElmer, Waltham, MA, USA). A quantitative analysis was performed by calculating the percentage of GFP-positive cells relative to the total cell number in each well.

### 2.10. MTS Assay

A549 cells (2.5 × 10^4^ cells/well of 96-well plate) were seeded and treated with different concentrations of PA as indicated for 24 h. Cell viability was assessed using a CellTiter 96^®^ AQueous One Solution Cell Proliferation Assay (MTS) kit (Promega Corporation, Madison, WI, USA) following the manufacturer’s protocol. In brief, 20 μL of the CellTiter 96^®^ Aqueous One Solution Reagent was added to each well of a 96-well plate, and the plate was incubated for 2 h at 37 °C. Absorbance was measured at 490 nm using a microplate reader, and cell viability was calculated based on the absorbance values.

### 2.11. Dual-Luciferase Reporter Assays

Dual-luciferase reporter assays were performed to measure ISRE promoter activity. HEK-293T (2 × 10^5^ cells/well of 12-well plate) cells were co-transfected with 500 ng of the ISRE-Luc reporter plasmid, 10 ng of the pRLTK plasmid (internal control), and 200 ng of the indicated plasmids or 200 ng empty vector using VigoFect transfection reagent (Vigorous). After 18 h of transfection, cells were treated with PA for an additional 8 h. Subsequently, cells were lysed with 1× passive lysis buffer (PLB) (Promega) for 15 min at room temperature with gentle shaking. The lysates were centrifuged to collect the supernatants, and firefly luciferase and Renilla luciferase activities were quantified using a Dual-Luciferase Reporter Assay System (Promega). Each experiment was independently repeated at least three times.

### 2.12. Quantification of cGAMP Using Enzyme-Linked Immunosorbent Assay (ELISA)

To quantify intracellular cGAMP levels, cells transfected with HT-DNA were washed twice with phosphate-buffered saline (PBS) and lysed using M-PER Mammalian Protein Extraction Reagent (ThermoFisher Scientific). The lysates were centrifuged at 14,000× *g* rpm for 20 min at 4 °C to remove cellular debris. The supernatants were collected, and cGAMP concentrations were measured using a commercially available cGAMP ELISA Kit (Cat# 501700, Cayman Chemical, Ann Arbor, MI, USA) following the manufacturer’s instructions.

### 2.13. Molecular Docking Simulation

The structures of RIG-I (PDB ID: 5E3H) and cGAS (PDB ID:4O68) were taken from the Protein Data Bank. The structure of PA (Compound CID: 5281125) was taken from PubChem. Molecular docking simulations were conducted by Discovery Studio2019 according to the manufacturer’s instructions.

### 2.14. ELISA

BMDM (1 × 10^6^ cells/well of 12-well plate) cells were transfected with 1 μg/mL of Poly(I:C) or HT-DNA for 12 h following PA pretreatment for 6 h. The secreted interferon in cell culture medium was analyzed with ELISA kits (439408, BioLegend, San Diego, CA, USA) according to the manufacturer’s instruction.

### 2.15. Statistical Analysis

Statistical analysis was performed using GraphPad Prism 10. Data are presented as the mean ± standard deviation (s.d.) or mean ± standard error of the mean (s.e.m.), as indicated in the figure legends. A two-tailed unpaired Student’s *t*-test was used to compare the two groups.

## 3. Results

### 3.1. Apiaceae-Family-Derived Petroselinic Acid Suppresses the Fluorescence of ISRE-GFP Reporter

**Figure 1:**
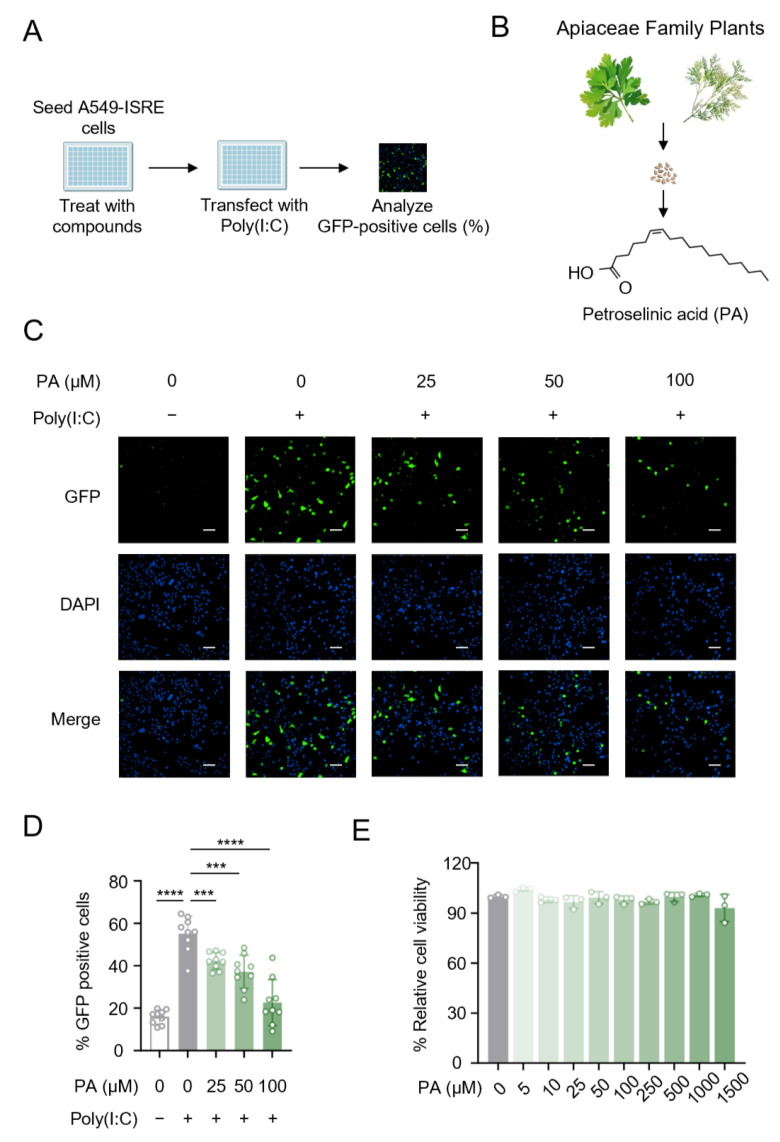
Apiaceae-family-derived petroselinic acid suppresses the fluorescence of ISRE-GFP reporter. (**A**) Schematic of experimental design for screening small-molecule inhibitors of type I IFN signaling. (**B**) The structural formula of petroselinic acid (PA) and its derivation. (**C**) Fluorescent micrographs by high-content of the ISRE-GFP reporter in human A549 cells treated with PA (0/25/50/100 μM) before transfection with 1 μg/mL poly(I:C). The white scale bar indicates a length of 30 μm. (**D**) The percentage of GFP positive cells in groups as indicated in (**C**). (**E**) Cell viability detection of A549 cells pretreated with different concentrations of PA as indicated. For (**D**), *p* values represent comparison with vehicle calculated using log rank test. All values are mean ± s.d.; *** *p* ≤ 0.001, **** *p* ≤ 0.0001; by unpaired *t*-test.

To screen for novel small-molecule inhibitors of type I IFN signaling, we established an ISRE reporter cell line stably expressing the ISRE-GFP reporter in human A549 cells. This system allowed for the capture of fluorescent signals via high-throughput screening upon the activation of type I IFN signaling and ISRE-GFP expression. Using this cell line, we screened a library of small molecules available in our laboratory to identify compounds that significantly modulate type I IFN signaling. Following pretreatment with candidate small molecules, A549 cells were transfected with poly(I:C), a synthetic double-stranded RNA (dsRNA), and the percentage of GFP-positive cells was quantified for statistical analysis (Figure 1A).

Among these potential candidate molecules used in our experiment, petroselinic acid (PA), a natural compound derived from Apiaceae family plants seeds (Figure 1B), was identified as a potent inhibitor of type I interferon signaling, and it demonstrated a concentration-dependent attenuation of ISRE-GFP reporter activity (Figure 1C,D). To evaluate the safety of PA, a toxicity testing assay was performed and cell viability was detected. Our findings revealed that PA does not induce significant cytotoxicity, even at high concentrations (up to 1500 µM), which are substantially above the effective inhibitory dose (50–100 µM) of PA (Figure 1E). Collectively, these data suggest that PA exerts an inhibitory effect on the fluorescence of the ISRE-GFP reporter, highlighting its potential as a negative modulator of type I IFN signaling.

### 3.2. Petroselinic Acid Suppresses Cytosolic-Nucleic-Acid-Induced IFN-I and ISG Expression

**Figure 2:**
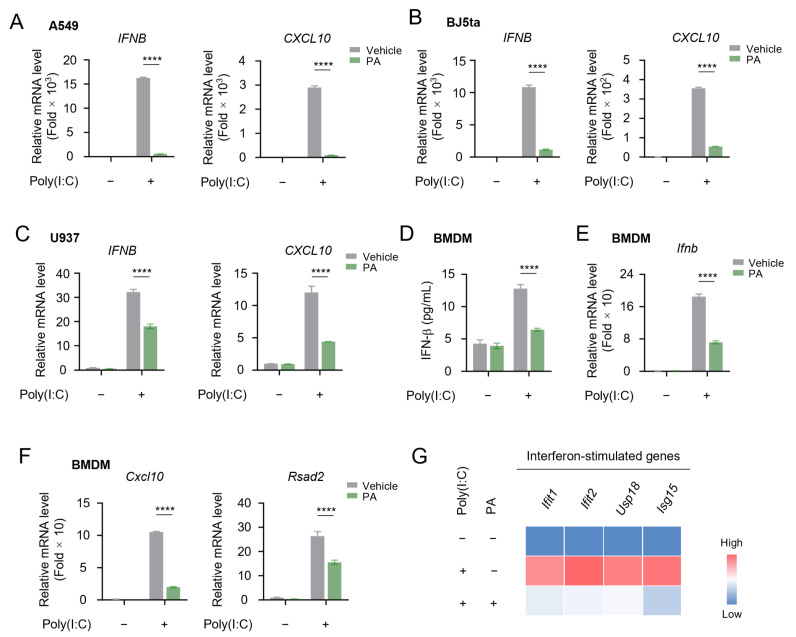
Petroselinic acid suppresses cytosolic-RNA-induced IFN-I and ISG expression. (**A**–**C**) RT-qPCR analysis of *IFNB* and *CXCL10* mRNA expression in A549 cells (**A**), BJ5ta cells (**B**), and U937 cells (**C**) transfected with 1 μg/mL poly(I:C) following 200 μM PA pretreatment for 6 h. (**D**) ELISA of secreted IFN-β in BMDM cells transfected with 1 μg/mL poly(I:C) following 200 μM PA pretreatment for 6 h. (**E**) RT-qPCR analysis of *Ifnb* mRNA expression in BMDM cells transfected with 1 μg/mL poly(I:C) following 200 μM PA pretreatment for 6 h. (**F**) RT-qPCR analysis of *Cxcl10* and *Rsad2* mRNA expression in BMDM cells transfected with 1 μg/mL poly(I:C) following 200 μM PA pretreatment for 6 h. (**G**) Heatmap of the levels of interferon-simulated genes (*Ifit1*, *Ifit2*, *Usp18*, *Isg15*) in BMDM cells transfected with 1 μg/mL poly(I:C) following 200 μM PA pretreatment for 6 h. For (**A**–**F**), *p* values represent comparison with vehicle calculated using log rank test. All values are mean ± s.d.; **** *p* ≤ 0.0001; by unpaired *t*-test.

To further elucidate the impact of petroselinic acid (PA) on the induction of type I IFN via the cytosolic RNA pathway, we validated its effects across various cell lines, including human non-immune cells, A549 (Figure 2A) and BJ5ta (Figure 2B), as well as the human immune cell line U937 (Figure 2C). In these different cell lines, PA significantly suppressed the expression of IFN-I and the downstream interferon-stimulated gene (ISG) *CXCL10* (Figure 2A–C). Moreover, we found that the cytosolic-dsRNA-induced IFN-β production and ISG expression in murine-bone-marrow-derived macrophages (BMDMs) were greatly attenuated (Figure 2D–G and Figure S1A–D).

**Figure 3:**
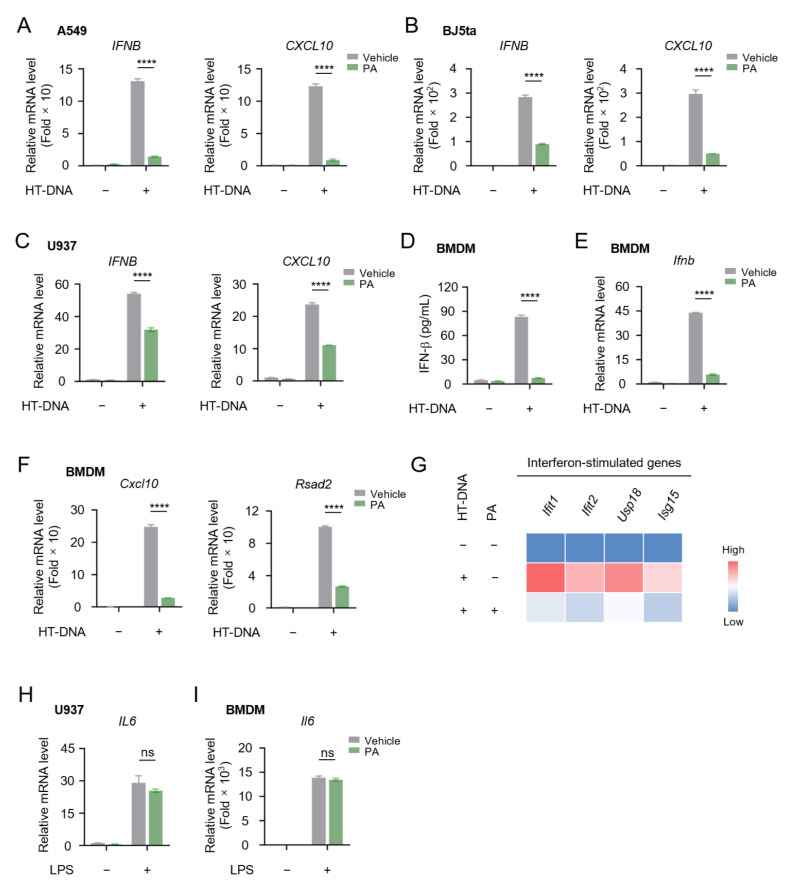
Petroselinic acid suppresses cytosolic-DNA-induced IFN-I and ISG expression. (**A**–**C**) RT-qPCR analysis of *IFNB* and *CXCL10* mRNA expression in A549 cells (**A**), BJ5ta cells (**B**), and U937 cells (**C**) transfected with 1 μg/mL HT-DNA following 200 μM PA pretreatment for 6 h. (**D**) ELISA of secreted IFN-β in BMDM cells transfected with 1 μg/mL HT-DNA following 200 μM PA pretreatment for 6 h. (**E**) RT-qPCR analysis of *Ifnb* mRNA expression in BMDM cells transfected with 1 μg/mL HT-DNA following 200 μM PA pretreatment for 6 h. (**F**) RT-qPCR analysis of *Cxcl10* and *Rsad2* mRNA expression in BMDM cells transfected with 1 μg/mL HT-DNA following 200 μM PA pretreatment for 6 h. (**G**) Heatmap of the levels of interferon-simulated genes (*Ifit1*, *Ifit2*, *Usp18*, *Isg15*) in BMDM cells transfected with 1 μg/mL HT-DNA following 200 μM PA pretreatment for 6 h. (**H**) RT-qPCR analysis of *IL6* mRNA expression in U937 cells stimulated with 1 μg/mL LPS following 200 μM PA pretreatment for 6 h. (**I**) RT-qPCR analysis of *Il6* mRNA expression in BMDM cells stimulated with 1 μg/mL LPS following 200 μM PA pretreatment for 6 h. For (**A**–**F**,**H**,**I**), *p* values represent comparison with vehicle calculated using log rank test. All values are mean ± s.d.; n.s., not significant; **** *p* ≤ 0.0001; by unpaired *t*-test.

In addition to cytosolic dsRNA, cytosolic DNA can also effectively activate the type I IFN pathway. Subsequently, we employed DNA mimics, herring testes DNA (HT-DNA), to activate interferon signaling and evaluated the influence of PA. Our data demonstrated that PA markedly inhibited the induction of IFN-I and downstream ISG *CXCL10* elicited by HT-DNA stimulation across various cell lines (Figure 3A–G and Figure S2A–D).

Apart from the type I IFN pathway triggered by cytosolic nucleic acids, we further explored whether PA could inhibit the TLR pathway, another important signaling in mediating innate immune responses. Using lipopolysaccharide (LPS) to activate TLR4, we observed a significant induction of pro-inflammatory cytokine *IL6*, which is triggered by NF-κB activation. However, PA failed to suppress *IL-6* production (Figure 3H,I). Taken together, our data indicated that PA specifically inhibits the cytosolic-nucleic-acid-induced type I IFN signaling pathway.

### 3.3. Petroselinic Acid Inhibits the Phosphorylation of TBK1 and IRF3 Induced by Cytosolic Nucleic Acids

**Figure 4:**
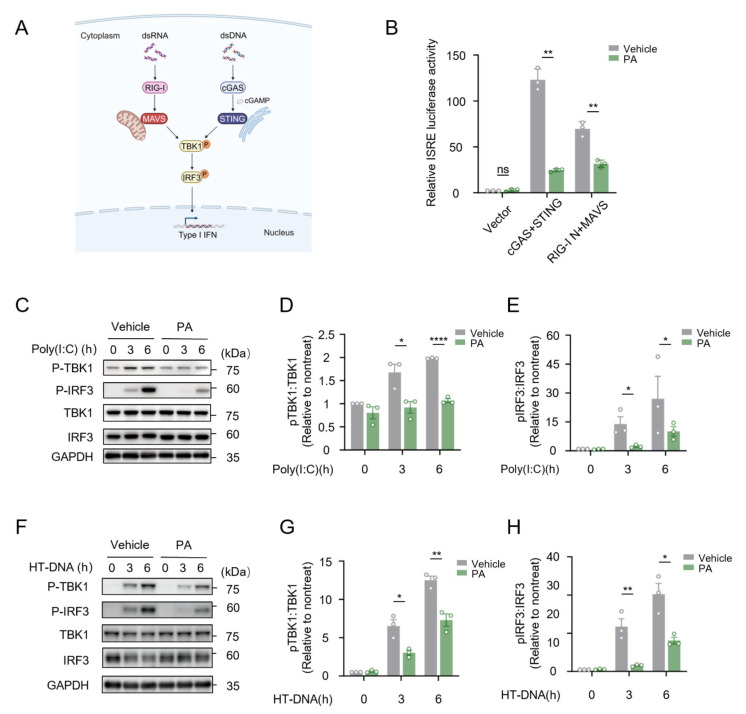
Petroselinic acid inhibits the phosphorylation of TBK1 and IRF3 induced by cytosolic nucleic acids. (**A**) Schematic of the type I IFN pathway induction by cytosolic nucleic acids. (**B**) Dual-luciferase reporter assays of ISRE reporter activation in HEK293T cells transfected with the indicated plasmids, followed by treatment with 200 μM PA. (**C**) Immunoblot assays of p-TBK1, p-IRF3, TBK1, and IRF3 in BJ5ta cells pretreated with 200 μM PA, and then transfected with 1 μg/mL poly(I:C). Sizes in kDa are indicated on the right. GAPDH was used as a loading control. (**D**,**E**) The intensities of p-TBK1 (**D**) and p-IRF3 (**E**) shown in (**C**) were quantified by Image J v1.53 (National Institutes of Health, Bethesda, MD, USA). (**F**) Immunoblot assays of p-TBK1, p-IRF3, TBK1, and IRF3 in BJ5ta cells pretreated with 200 μM PA, and then transfected with 1 μg/mL HT-DNA. Sizes in kDa are indicated on the right. GAPDH was used as a loading control. (**G**,**H**) The intensities of p-TBK1 (**G**) and p-IRF3 (**H**) shown in (**F**) were quantified by Image J. For (**B**,**D**,**E**,**G**,**H**), *p* values represent the comparison with the vehicle calculated using a log rank test. All values are mean ± s.d.; n.s., not significant; * *p* ≤ 0.05, ** *p* ≤ 0.01, **** *p* ≤ 0.0001; by unpaired *t*-test. Original Western blot images of (**C**,**F**) can be found in Figure S4.

Cytosolic dsRNA is primarily sensed by RIG-I-like Receptors (RLRs). The recognition of dsRNA by RLRs triggers its recruitment of MAVS, a mitochondrion-associated adapter protein. In contrast, cytosolic DNA is sensed by cGAS. Upon detection of DNA, cGAS synthesizes cGAMP using GTP and ATP as substrates, which in turn binds to the ER-located adapter protein STING. Activated MAVS or STING subsequently recruits TBK1 and IRF3, leading to their phosphorylation and induction of IFN-I and downstream ISG expression (Figure 4A).

To investigate whether PA could regulate RLRs or cGAS-induced type I IFN activation, RIG-I/MAVS or cGAS/STING plasmids were co-transfected with an ISRE luciferase reporter plasmid into HEK293T cells prior to PA treatment. At 24 h post-transfection, cells were subjected to dual-luciferase reporter assays to detect the promoter activity of the ISRE. Consistent with our previous findings, PA significantly inhibited ISRE activation driven by RIG-I/MAVS or cGAS/STING (Figure 4B). However, when HEK 293T cells were transfected with MDA5/MAVS, PA failed to inhibit the luciferase activity of the ISRE reporter (Supplementary Figure S3), suggesting that PA has specificity for the RIG-I-mediated recognition of cytosolic RNA in the activation of the type I interferon signaling pathway.

We next elucidated the detailed mechanism by which PA inhibits the cytosolic nucleic acids sensing pathway. Since TBK1 and IRF3 phosphorylation are key steps for RIG-I and cGAS mediated type I IFN activation, we next detected whether PA could affect these crucial events. Our results showed that PA treatment markedly suppressed the phosphorylation of TBK1 and IRF3 in response to the poly(I:C) (Figure 4C–E) and HT-DNA challenge (Figure 4F–H). These findings further supported our conclusion that PA specifically inhibits cytosolic-nucleic-acid-mediated the type I interferon signaling pathway, working at TBK1 or its upstream level.

### 3.4. Petroselinic Acid Functions as a Binding Partner of Cytosolic Nucleic Acid Sensor Proteins cGAS and RIG-I

**Figure 5:**
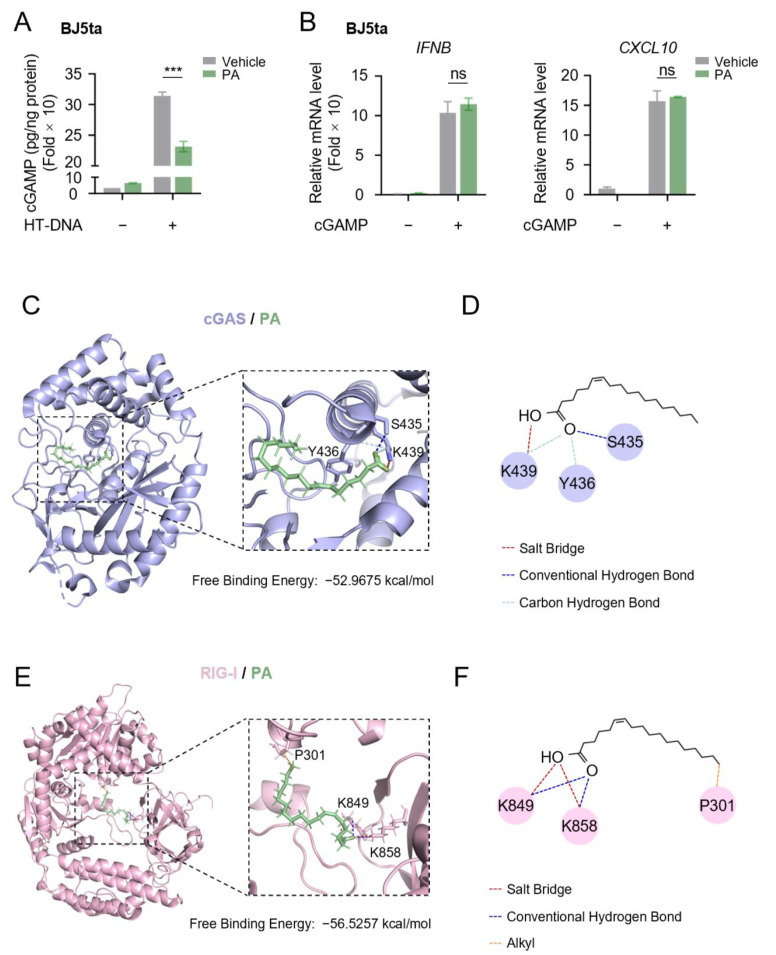
Petroselinic acid functions as a binding partner of cytosolic nucleic acids sensor proteins cGAS and RIG-I. (**A**) ELISA analysis of cGAMP production in BJ5ta cells transfected with 1 μg/mL HT-DNA following 200 μM PA pretreatment for 6 h. (**B**) RT-qPCR analysis of *IFNB* and *CXCL10* mRNA expression in BJ5ta cells stimulated with 2 μg/mL cGAMP following 200 μM PA pretreatment for 6 h. (**C**) Molecular docking model of PA bound to cGAS. Ribbon view of the cGAS/PA binding pocket. Amino acid residues involved in PA binding in cGAS are labelled. (**D**) Two-dimensional representation of interactions between cGAS and PA. (**E**) Molecular docking model of PA bound to RIG-I. Ribbon view of the RIG-I/PA binding pocket. Amino acid residues involved in PA binding in RIG-I are labelled. (**F**) Two-dimensional representation of interactions between RIG-I and PA. For (**A**,**B**), *p* values represent the comparison with the vehicle calculated using a log rank test. All values are mean ± s.d.; n.s., not significant; *** *p* ≤ 0.001; by unpaired *t*-test.

To further study the effect of PA on type I IFN signaling, we next investigated its impact on cGAMP synthesis after HT-DNA stimulation. Interestingly, the results revealed that PA significantly inhibits cGAMP production (Figure 5A), indicating it may affect cGAS activity. Consistently, PA failed to inhibit *IFNB* and *CXCL10* expression induced by cGAMP (Figure 5B). These findings suggested that PA may exert its effects on sensor proteins.

To validate this hypothesis, we conducted molecular docking simulations of PA with the sensor proteins cGAS and RIG-I. Remarkably, we observed that PA interacts with cGAS with a binding free energy of −52.9675 kcal/mol (Figure 5C), forming a conventional hydrogen bond with Ser435, carbon hydrogen bonds with Tyr436 and Lys439, and a salt bridge with Lys439 (Figure 5D). Additionally, we found PA could also bind to the RNA sensor RIG-I with a binding free energy of −56.5257 kcal/mol (Figure 5E), forming an alkyl bond with Pro301, and salt bridges as well as conventional hydrogen bonds between Lys848 and Lys858 (Figure 5F). Overall, these results indicate that PA could directly interact with the sensor proteins cGAS and RIG-I to inhibit their activation, thereby attenuating the interferon signaling pathway.

### 3.5. Petroselinic Acid Ameliorates Cytosolic-Nucleic-Acid-Mediated Autoimmune Disorders

**Figure 6:**
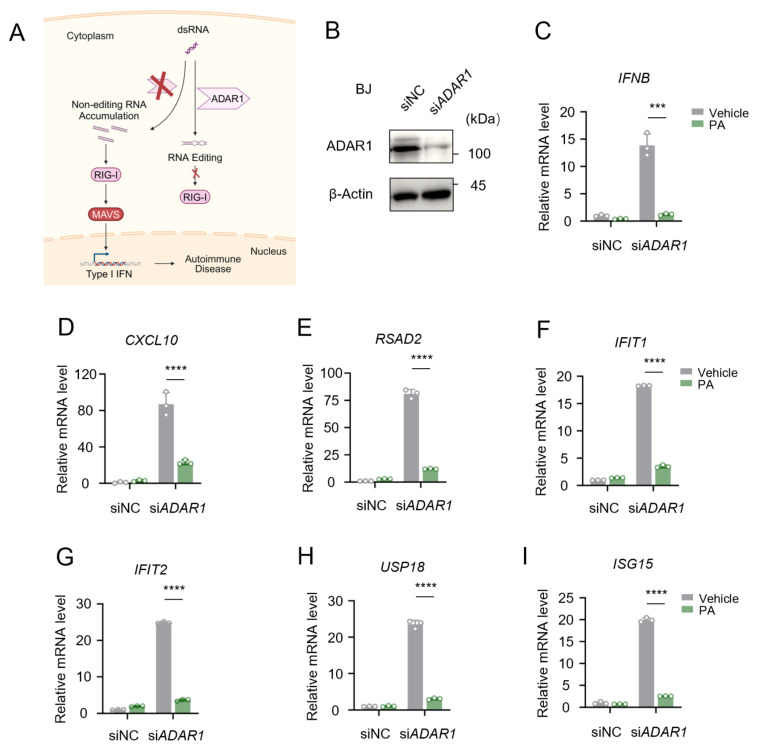
Petroselinic acid ameliorates cytosolic-RNA-mediated autoimmune disorders. (**A**) Schematic of the pathogenesis of AGS following mutation in the *ADAR1* gene. (**B**) Immunoblot assays of ADAR1 in BJ5ta cells transfected with siNC or *ADAR1*-specific siRNA (si*ADAR1*). Sizes in kDa are indicated on the right. β-Actin was used as a loading control. (**C**–**I**) RT-qPCR analysis of *IFNB* (**C**), *CXCL10* (**D**), *RSAD2* (**E**), *IFIT1* (**F**), *IFIT2* (**G**), *USP18* (**H**), and *ISG15* (**I**) mRNA expression in BJ5ta cells following 200 μM PA pretreatment for 24 h. For (**C**–**I**), *p* values represent the comparison with the vehicle calculated using a log rank test. All values are mean ± s.d.; *** *p* ≤ 0.001, **** *p* ≤ 0.0001; by unpaired *t*-test. Original Western blot image of (**B**) can be found in Figure S5.

It is well established that the aberrant and excessive activation of the type I IFN pathway by cytosolic nucleic acids can lead to autoimmune diseases. Aicardi–Goutières syndrome (AGS) is a rare monogenic autoimmune disease primarily caused by gene mutations that result in the accumulation of self-nucleic acids in the cytoplasm or the aberrant sensing of self-nucleic acids. This condition triggers the overproduction of type I IFN and downstream ISGs, ultimately leading to AGS. The RNA-editing enzyme ADAR1 (Adenosine Deaminase Acting on RNA 1) is pivotal in preventing autoimmune disease by curbing the accumulation of non-edited immunostimulatory double-stranded RNA. Mutations in ADAR1 are closely associated with the development of AGS (Figure 6A). To investigate the potential therapeutic benefits of PA on ADAR1-associated AGS, we knocked down *ADAR1* in BJ5ta cells (Figure 6B). This manipulation led to the abnormal accumulation of dsRNA in cytoplasm, triggering the sustained activation of the type I interferon signaling pathway. Notably, pretreatment with PA significantly suppressed the expression of *IFNB* and several ISGs, including *CXCL10*, *RSAD2*, *IFIT1*, *IFIT2*, *USP18*, and *ISG15* (Figure 6C–I). These findings suggest that PA ameliorates cytosolic-dsRNA-mediated autoimmune responses.

**Figure 7:**
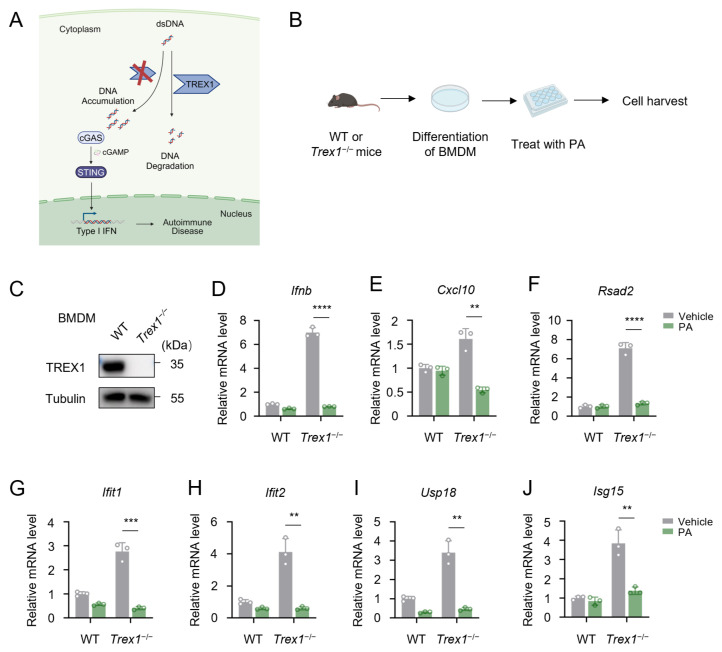
Petroselinic acid ameliorates cytosolic-DNA-mediated autoimmune disorders. (**A**) Schematic of the pathogenesis of AGS following mutation in the *TREX1* gene. (**B**) Schematic of experiments for bone-marrow-derived macrophages (BMDMs) from *Trex1*^−/−^ mice treated with or without PA. (**C**) Immunoblot assays of TREX1 in BMDM cells from wild-type and *Trex1*^−/−^ mice. Sizes in kDa are indicated on the right. Tubulin was used as a loading control. (**D**–**J**) RT-qPCR analysis of *Ifnb* (**D**), *Cxcl10* (**E**), *Rsad2* (**F**), *Ifit1* (**G**), *Ifit2* (**H**), *Usp18* (**I**), and *Isg15* (**J**) mRNA expression in BMDM cells from wild-type and *Trex1*^−/−^ mice following 200 μM PA pretreatment for 12 h. For (**D**–**J**), *p* values represent the comparison with the vehicle calculated using a log rank test. All values are mean ± s.e.m.; ** *p* ≤ 0.01, *** *p* ≤ 0.001, **** *p* ≤ 0.0001; by unpaired *t*-test. Original Western blot image of (**C**) can be found in Figure S6.

The DNA 3′ repair exonuclease TREX1 (three prime repair exonuclease 1) is always responsible for the degradation of cytosolic DNA, and its mutations also lead to AGS (Figure 7A). *Trex1*^−/−^ mice displayed severe autoimmune responses similar to those observed in patients with AGS. We next explored whether PA could be effective in treating cytosolic-DNA-mediated autoimmune disorders. We isolated bone-marrow-derived macrophages from both wild-type and *Trex1*^−/−^ mice (Figure 7B). In *Trex1*^−/−^ BMDM cells, the level of TREX1 protein was significantly reduced (Figure 7C). Compared with wild-type cells, the *Trex1*^−/−^ BMDM cells showed markedly elevated expressions of *Ifnb* and ISGs (Figure 7D–J). Treatment with PA significantly reduced the level of *Ifnb* and ISGs in *Trex1*^−/−^ BMDM cells (Figure 7D–J). These findings collectively demonstrated that PA exerts a potent inhibitory effect on autoimmune diseases induced by abnormal cytosolic nucleic acids.

## 4. Discussion

Cytosolic nucleic acid recognition is a cornerstone of the innate immune system, serving as a critical mechanism in initiating type-I-IFN-mediated immune responses [11,21]. PRRs, such as RIG-I, MDA5, and cGAS, play pivotal roles in detecting cytosolic dsRNA and DNA, which may originate from either pathogens or damaged tissue [2,22,23]. Upon the detection of cytosolic nucleic acids, these sensors activate signaling cascades that ultimately lead to the production of type I IFN [23]. Although these processes are essential for immune defense and maintaining homeostasis, their dysregulation can lead to profound pathological outcomes. Specifically, the sustained activation of cytosolic nucleic acid sensing pathways, often driven by the abnormal accumulation of cytosolic nucleic acids, can result in excessive type I IFN production. This dysregulated response has been implicated in a variety of pathological conditions, including chronic inflammation, cellular senescence, and autoimmune disorders [4,24].

The pathogenic role of excessive type I IFN signaling is particularly evident in diseases such as Aicardi–Goutières Syndrome (AGS), where mutations in key nucleic acid regulatory proteins, such as TREX1 and ADAR1, lead to the aberrant accumulation of cytosolic nucleic acids [25,26]. This triggers a hyperactive immune response, characterized by the overexpression of type I IFN and its downstream ISGs, ultimately contributing to tissue damage and systemic autoimmunity [27]. These findings underscore the delicate balance between immune activation and immune tolerance, emphasizing the importance of maintaining immune homeostasis to prevent pathological immune responses.

In light of these well-established observations, therapeutic strategies aimed at suppressing excessive type I IFN signaling represent a promising avenue for mitigating autoimmune disorders [28]. For instance, some chemical inhibitors targeting key components of the nucleic acid sensing pathways have shown potential in preclinical models [29]. Additionally, agents capable of reducing the accumulation of cytosolic nucleic acids may offer alternative approaches to restoring immune homeostasis [30]. Notably, the inhibition of interferon activation using natural compounds isolated from various plants has emerged as a promising approach to alleviate autoimmune diseases. Apart from EGCG isolated from green tea and curcumin from turmeric, other small molecules such as quercetin and sulforaphane, derived from various fruits and vegetables, have demonstrated potential activities in modulating type I interferon signaling and related immune diseases [14,15,31,32]. After systematic screening, we identified several candidates with potential roles in type I IFN signaling. PA emerged as a prioritized compound due to its minimal cytotoxicity, robust dose-dependent effects, and natural origination from plants. This combination of properties suggests potential practical applications and thereby has garnered our particular interest. Our study revealed that PA, a monounsaturated fatty acid derived from Apiaceae family plants, exerts significant inhibitory effects on interferon production induced by cytosolic nucleic acids. PA’s ability to suppress IFN production also has important implications for the treatment of specific autoimmune diseases, such as AGS. These findings offer a novel therapeutic strategy for treating autoimmune diseases or inflammatory damage by targeting IFN-related pathways.

Certain members of the Apiaceae family plants have long been recognized for their potential anti-inflammatory and antioxidant properties, while the involvement of PA in innate immune regulation, particularly in modulating cytosolic-nucleic-acid-mediated interferon production, remains elusive [33,34]. Despite growing evidence supporting its beneficial effects, the underlying mechanisms governing these activities are poorly understood. Our findings revealed that PA exerts a profound impact on innate immune activation triggered by cytosolic nucleic acids, particularly in the context of type I IFN responses. This study expanded the understanding of PA’s immune-modulatory functions by elucidating its crucial role in regulating IFN production and its downstream signaling. Importantly, we found that PA could directly interact with the sensor proteins cGAS and RIG-I and inhibit the phosphorylation of TBK1 and IRF3 in response to cytosolic DNA and RNA challenges. Regarding how PA interacts with sensor proteins and affects their functions, we compared PA with known sensor proteins inhibitors [35,36,37,38,39] through an analysis of known crystal structure and molecular docking simulations (Tables S1 and S2). Our findings suggested that PA may competitively bind to the ATP binding site on cGAS, subsequently inhibiting its enzymatic activity [40]. Additionally, PA may exert its inhibitory effect on RIG-I by occupying the RNA recognition site, thereby disrupting its signaling function [41].

In the context of small fatty-acid-based molecules, several structurally analogous compounds have been reported to exhibit comparable immune-modulatory effects. For instance, oleic acid, a monounsaturated fatty acid, has been shown to inhibit cGAS-DNA phase separation, consequently suppressing the production of cGAMP and interferon, while leaving the RNA pathway unaffected [42]. Another case is rosmarinic acid, which can be isolated from plants of the Apiaceae family. Studies have proven that it can suppress the activation of the cytosolic nucleic acid sensing interferon pathway [43]. In contrast, certain polyunsaturated fatty acids have been demonstrated to promote the cytosolic nucleic acid pathway, highlighting the diverse roles of fatty acids in immune regulation [44]. These findings underscore the complexity of fatty acid interactions with the immune system and suggest that PA’s effects on innate immunity may be part of a broader pattern observed among fatty-acid-derived molecules. Our study adds to this body of knowledge by providing detailed insights into PA’s direct interactions with key immune sensors and its downstream effects on signaling pathways. These results position PA as a promising candidate for further exploration in immune-modulatory therapies, paving the way for innovative strategies to address immune dysregulation.

While our findings highlight the significant potential of PA in immune modulation and IFN regulation, several challenges remain to be further investigated. For example, the precise mechanisms underlying PA impaction on cGAS and RIG-I sensors are not fully clarified. Further biophysical methods, such as surface plasmon resonance (SPR), should be employed to validate the direct interaction between PA and sensor proteins. Although we demonstrated that PA significantly mitigates interferon and ISG production in ADAR1- and Trex1-related AGS models, substantial efforts should be undertaken to examine the effect of PA on AGS and other autoimmune diseases in vivo. Moreover, the clinical application of PA also requires further exploration. As a natural small molecule, PA requires a relatively higher effective concentration compared to other chemically synthesized small-molecule inhibitors [35,36,38]. This implies that for its practical application, further chemical modification of PA may be necessary in future research. The excessive activation of nucleic acid sensing signaling and the release of IFN-associated inflammatory cytokines, usually triggered by host-derived nucleic acid fragments in the absence of infection, can lead to sustained tissue inflammation and damage, contributing to the development of senescence [24,45]. As a critical negative regulator, PA effectively suppresses the overactivation of the IFN pathway. Whether PA could contribute to reduce tissue inflammation or damage, even slow cellular senescence processes, warrants further investigation.

## 5. Conclusions

In conclusion, this study identified petroselinic acid (PA), a natural compound from Apiaceae plants, as a potential inhibitor of cytosolic-nucleic-acid-induced interferon production. Our findings significantly expand the functional repertoire of PA by revealing its intricate mechanisms of immune regulation and IFN modulation. Furthermore, by highlighting its versatility in treating interferon-related immune disorders, this study paves the way for developing PA as a therapeutic option for a broad spectrum of inflammatory and autoimmune conditions.

## Data Availability

All data can be obtained from the corresponding authors upon reasonable request.

## Supplementary Materials

The following supporting information can be downloaded at: https://www.mdpi.com/article/10.3390/biom15030329/s1, Figure S1: Petroselinic acid suppresses cytosolic RNA-induced ISGs expression; Figure S2: Petroselinic acid suppresses cytosolic DNA-induced ISGs expression; Figure S3: Petroselinic acid fails to inhibit ISRE activation driven by MDA5/MAVS; Figure S4: Original Western blot images of Figure 4C,F; Figure S5: Original Western blot images of Figure 6B; Figure S6: Original Western blot images of Figure 7C; Table S1: Free binding energy and interacting amino acids for each inhibitor to cGAS; Table S2: Free binding energy and interacting amino acids for each inhibitor to RIG-I.
