# Supplementary material for: Petroselinic Acid from Apiaceae Family Plants Ameliorates Autoimmune Disorders Through Suppressing Cytosolic-Nucleic-Acid-Mediated Type I Interferon Signaling
Source: Biomolecules. 2025 Feb 24;15(3):329. doi: 10.3390/biom15030329 (PMC11939978; doi:10.3390/biom15030329)
Supplement: Supplementary file 1 [file biomolecules-15-00329-s001.zip › 20250224 Original Images.pdf]

Figure1.C-D

−Poly(I:C) −PA

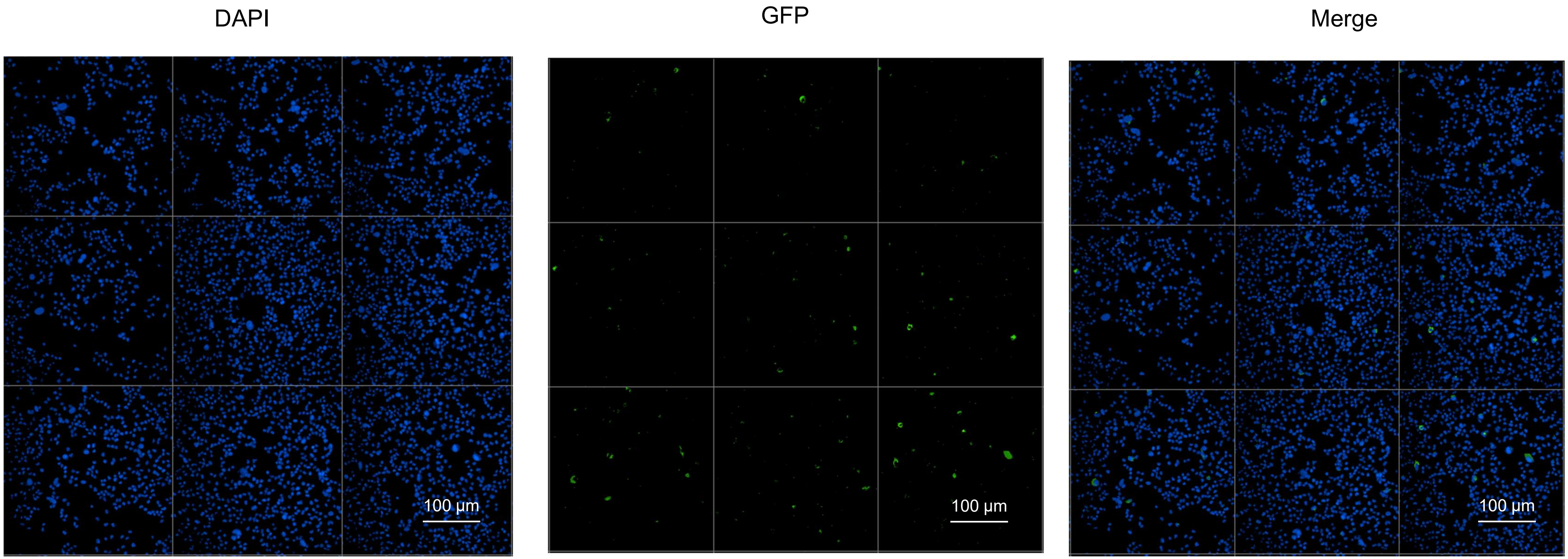

Figure1.C-D

+Poly(I:C) −PA

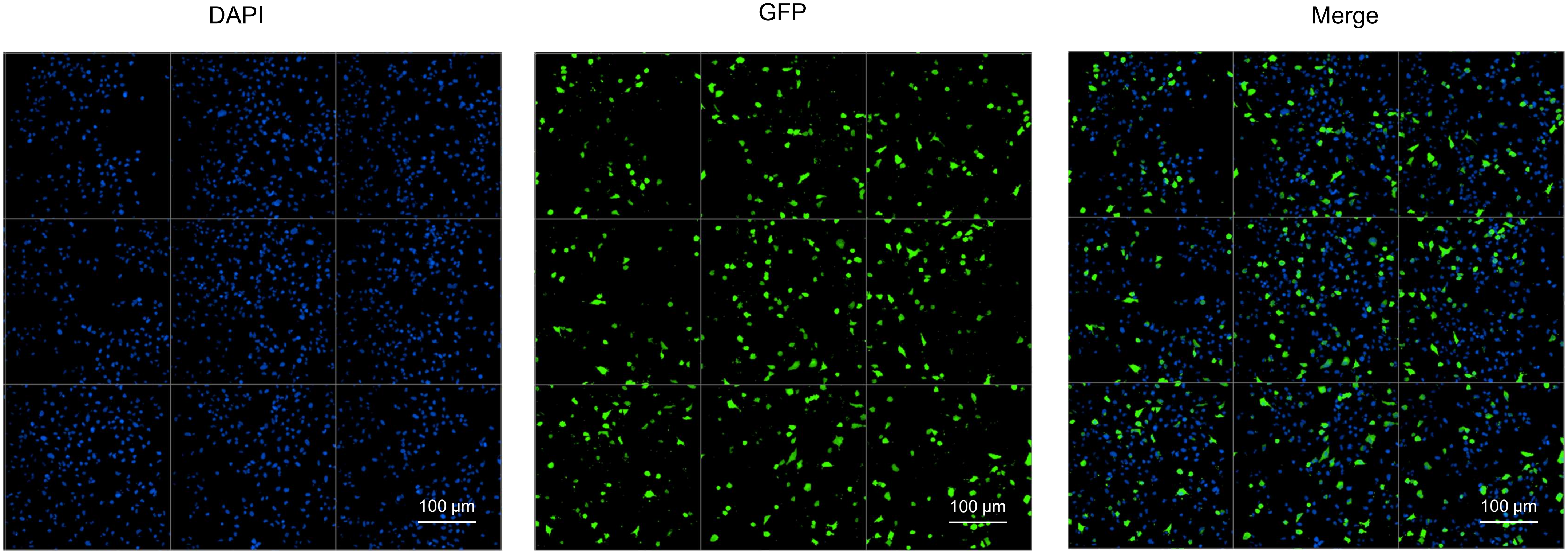

Figure1.C-D

+Poly(I:C) PA 25μM

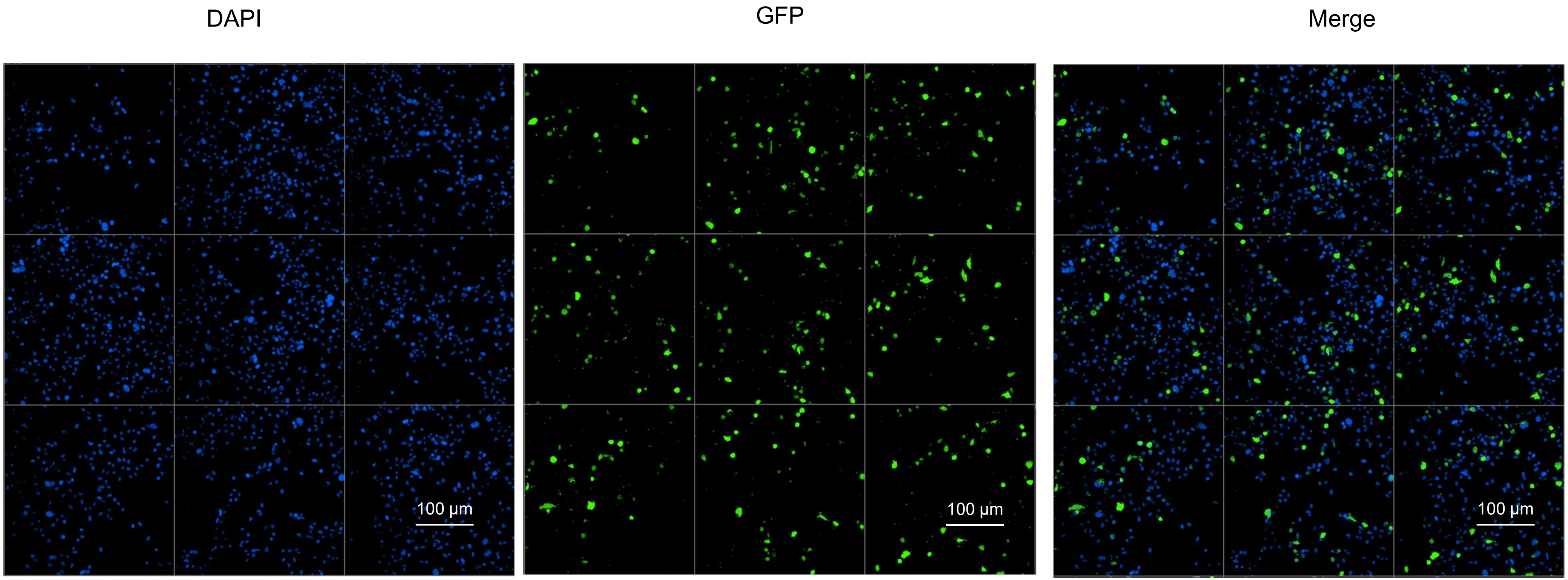

Figure1.C-D

+Poly(I:C) PA 50μM

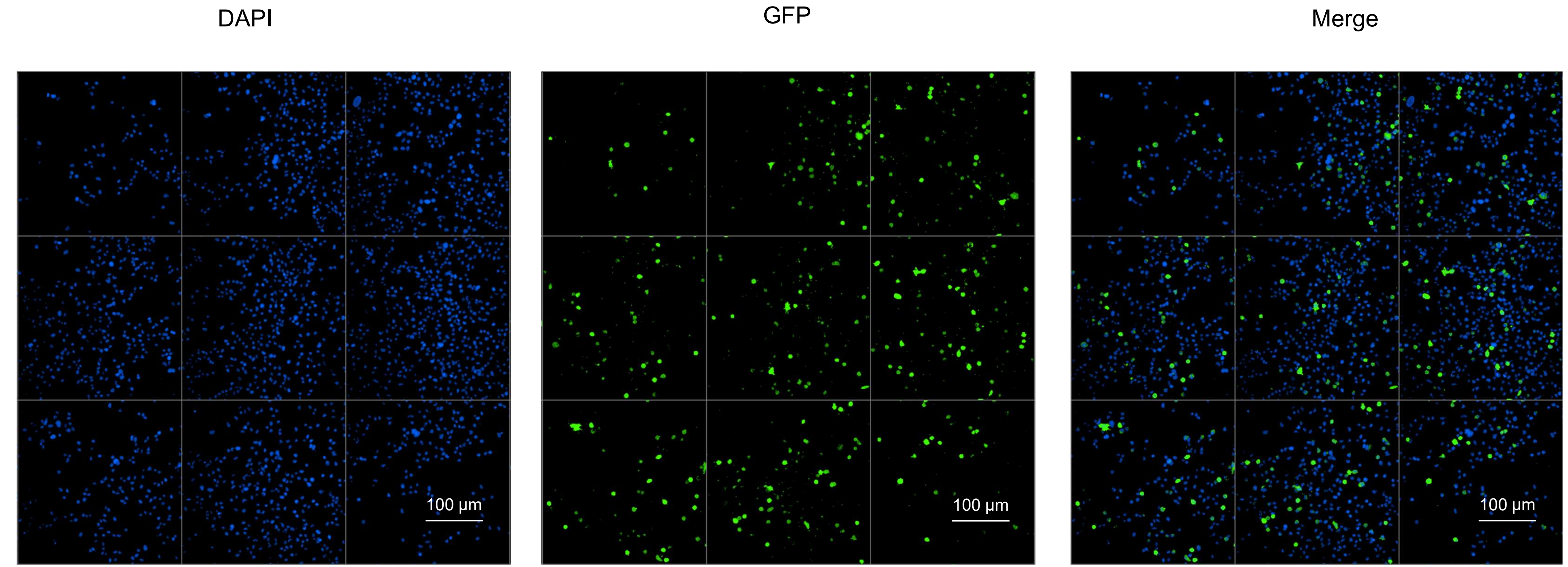

Figure1.C-D

+Poly(I:C) PA 100μM

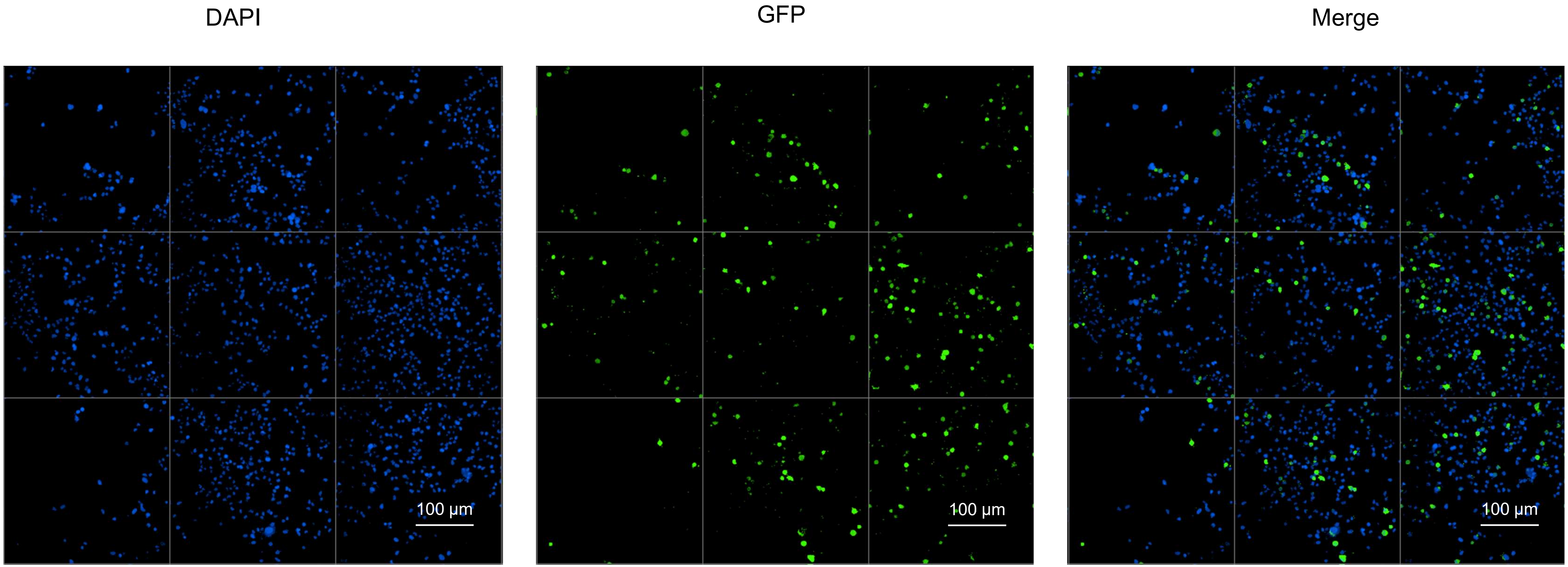

Figure4.C-E

Duplication1

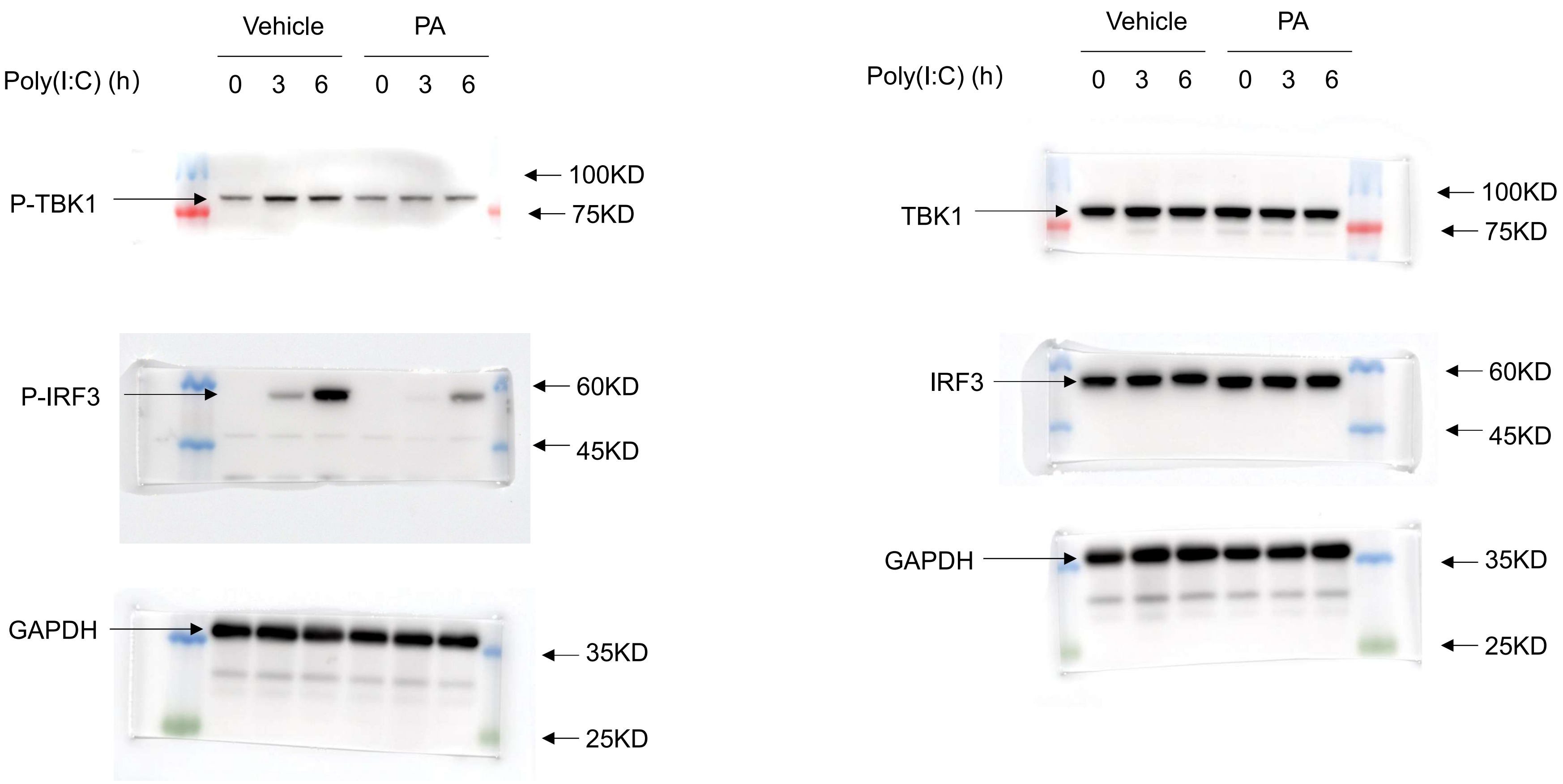

Figure4.C-E

Duplication2

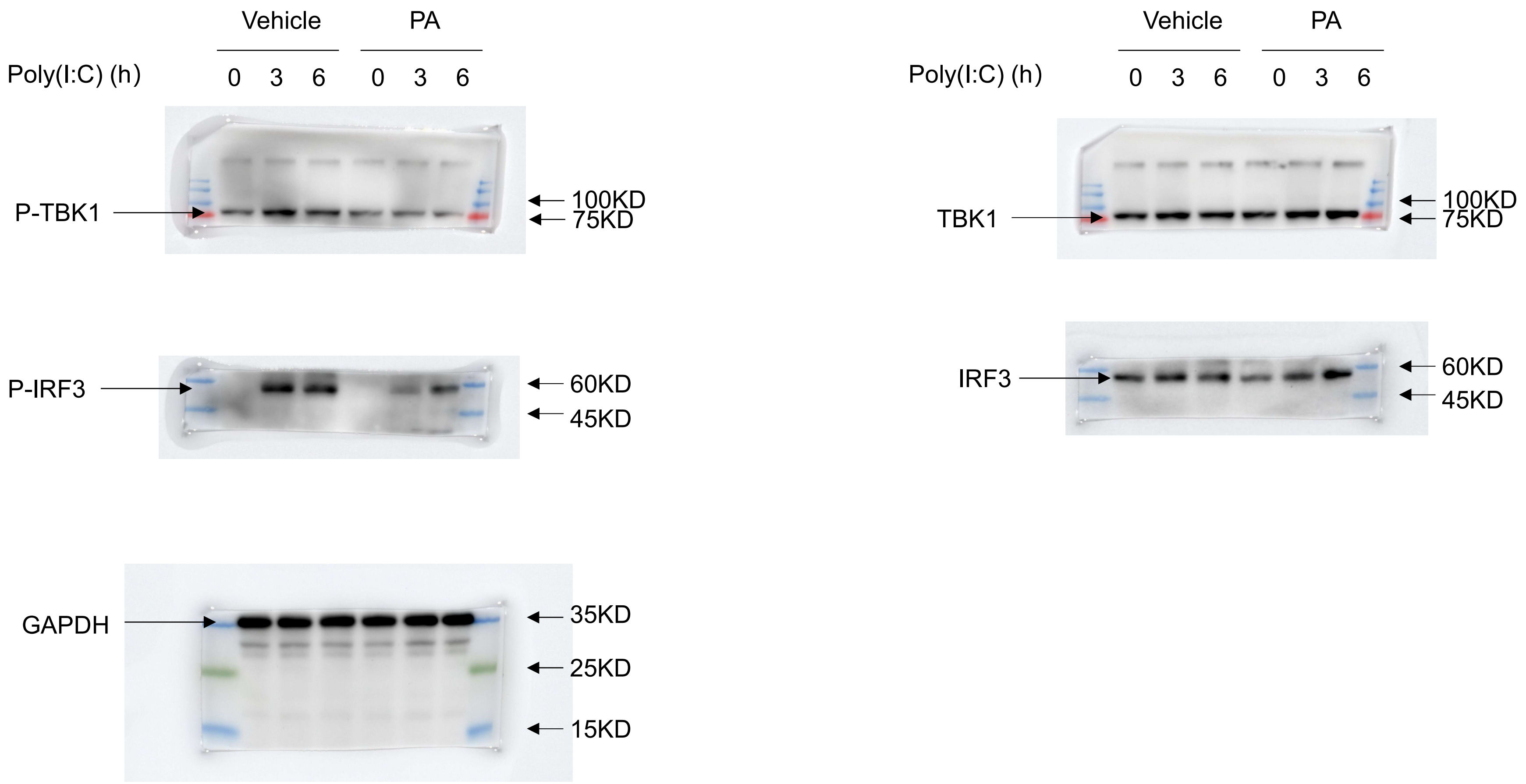

Figure4.C-E

Duplication3

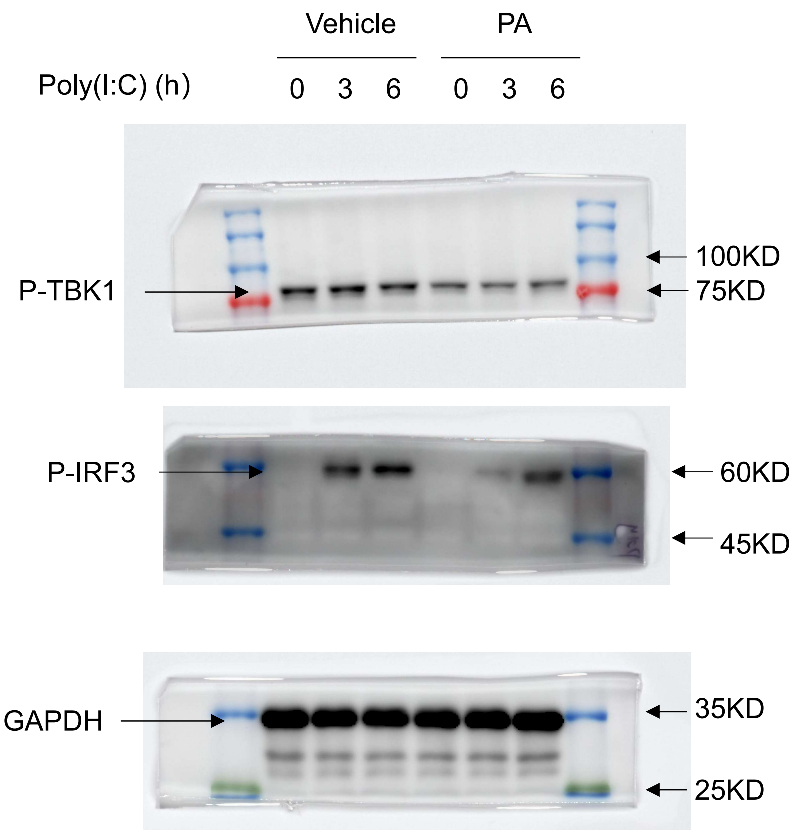

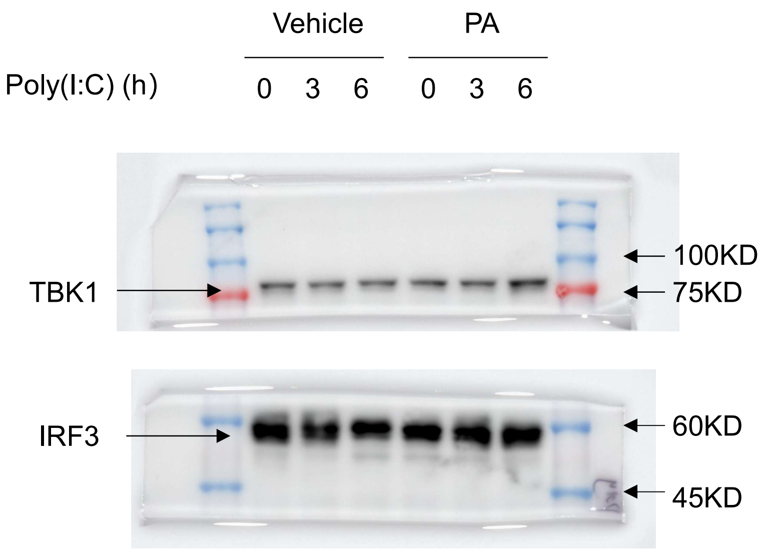

Figure4.F-H

Duplication1

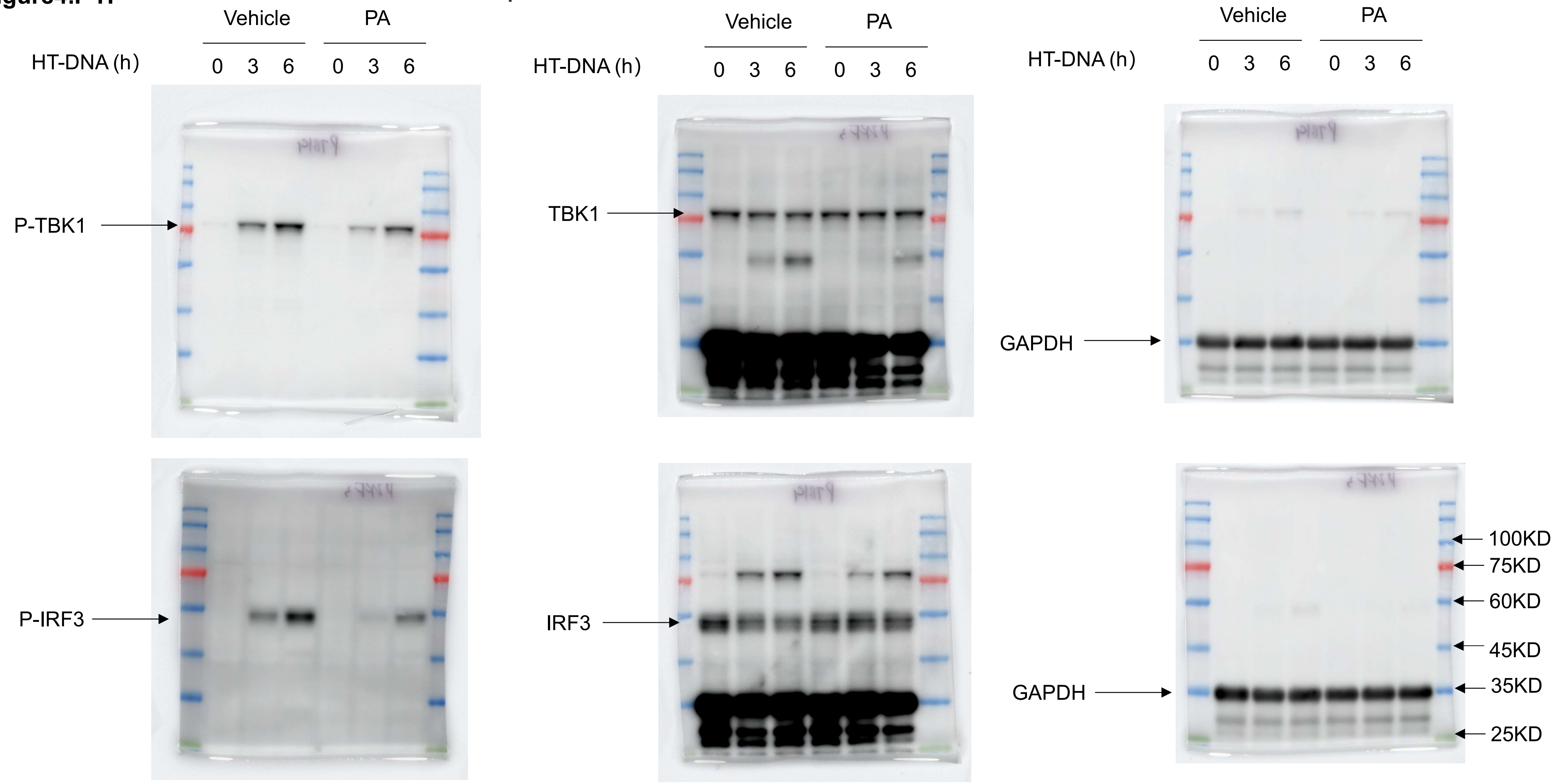

Figure4.F-H

Duplication2

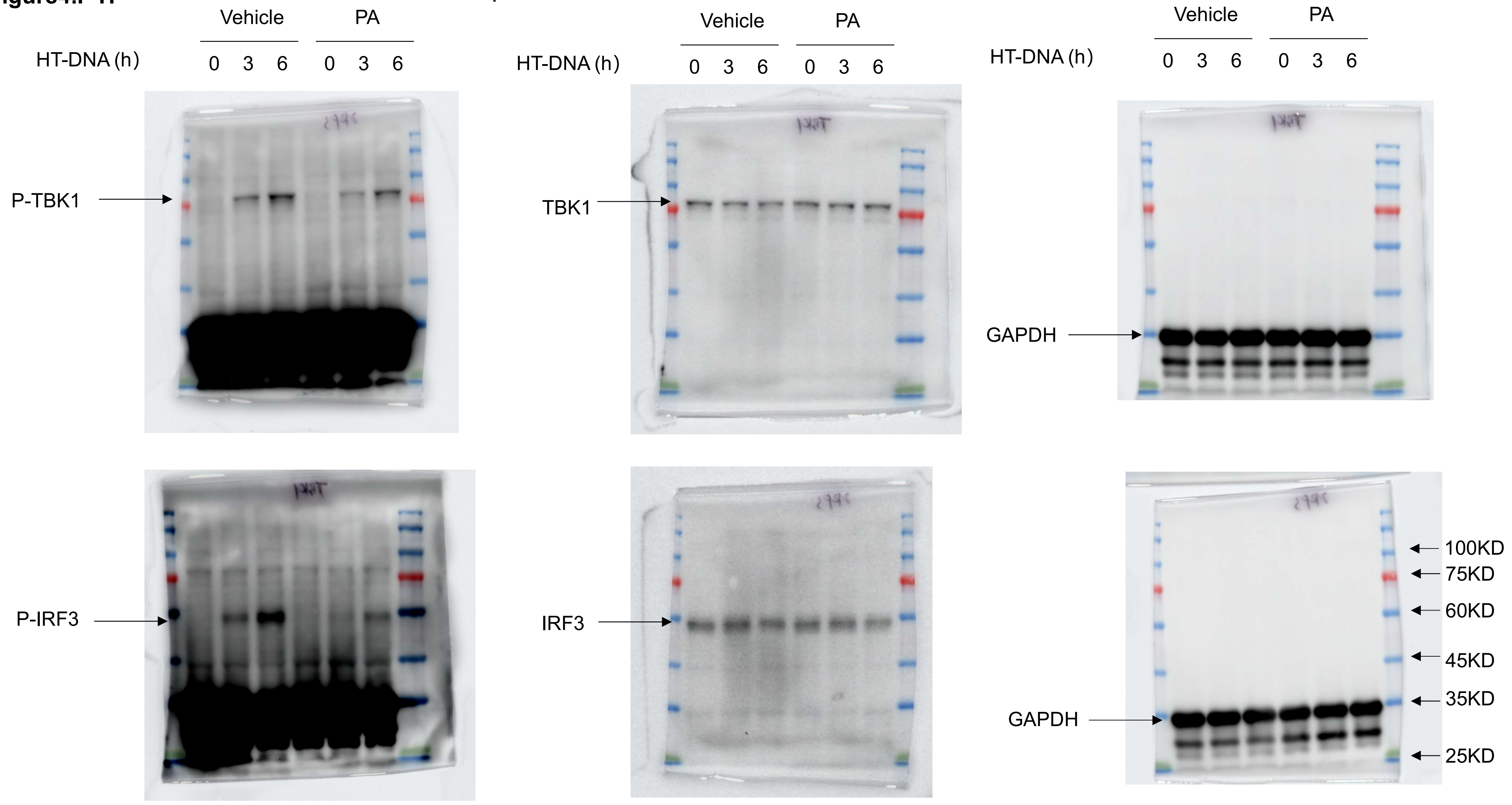

Figure4.F-H

Duplication3

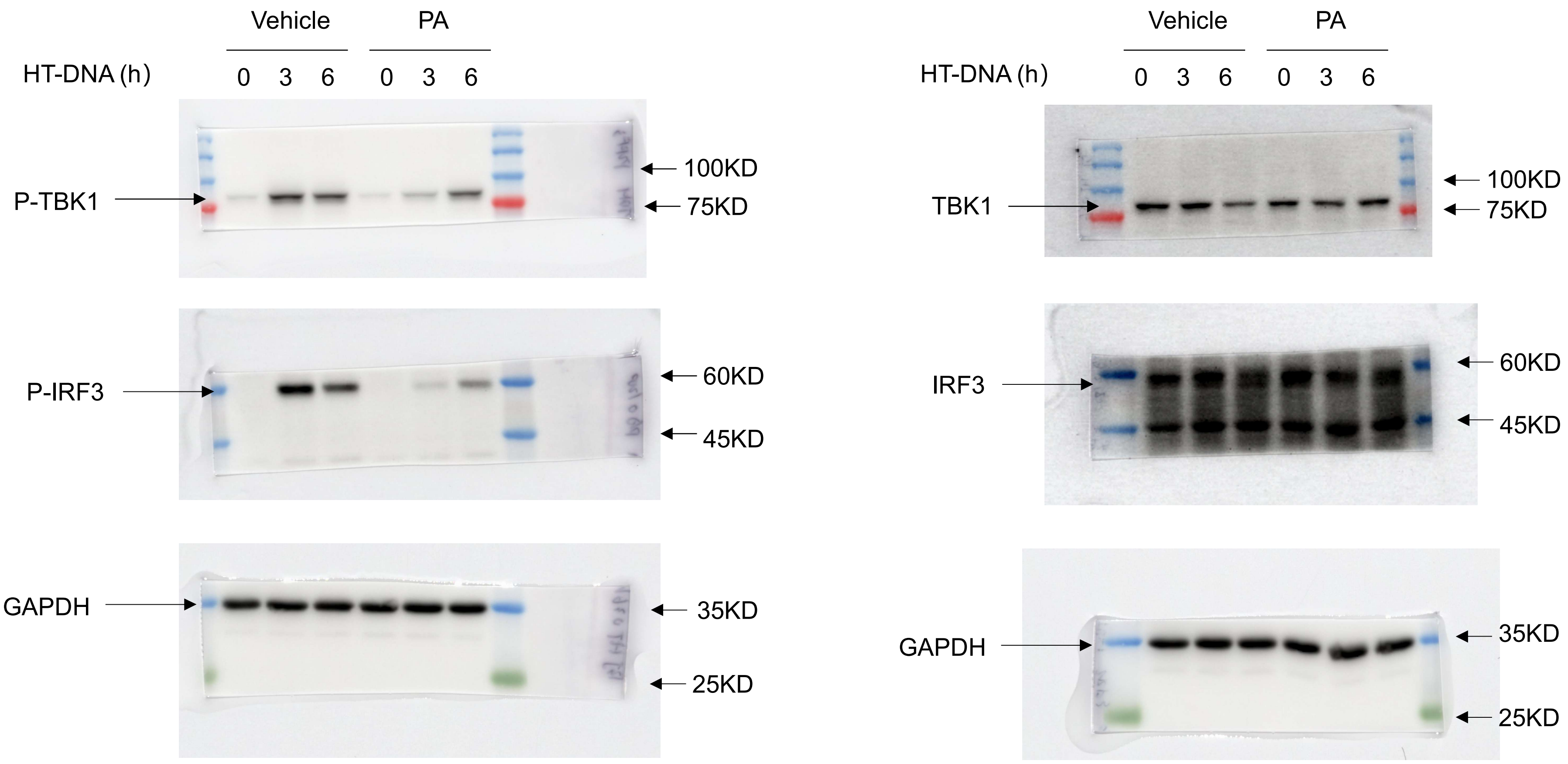

Figure6.B

BJ siADAR1 WB TEST

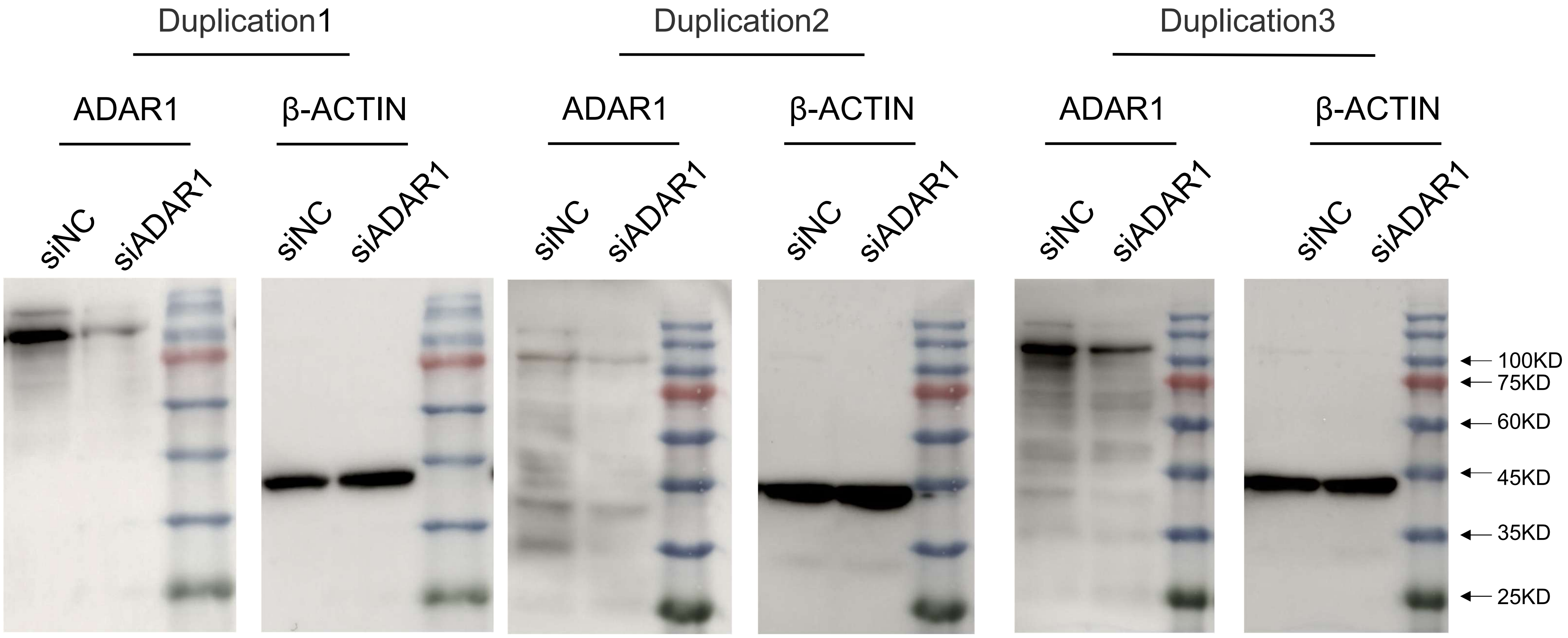

Figure7.C

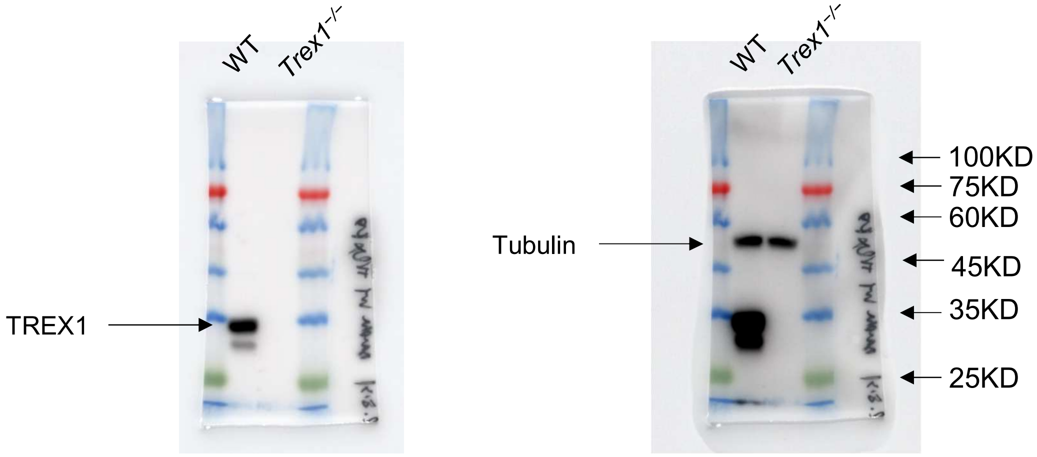
WT
$Trex1^{-/-}$
TREX1
WT
$Trex1^{-/-}$
Tubulin
100KD
75KD
60KD
45KD
35KD
25KD
