# Supplementary material for: Petroselinic Acid from Apiaceae Family Plants Ameliorates Autoimmune Disorders Through Suppressing Cytosolic-Nucleic-Acid-Mediated Type I Interferon Signaling
Source: Biomolecules. 2025 Feb 24;15(3):329. doi: 10.3390/biom15030329 (PMC11939978; doi:10.3390/biom15030329)
Supplement: Supplementary file 1 [file biomolecules-15-00329-s001.zip › 20250224 Supplementary Figure.pdf]

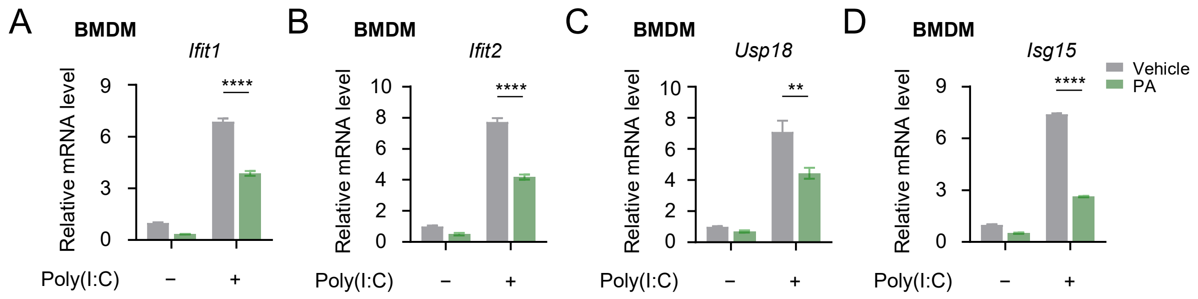

Supplementary Figure 1. Petroselinic acid suppresses cytosolic RNA-induced ISGs expression.

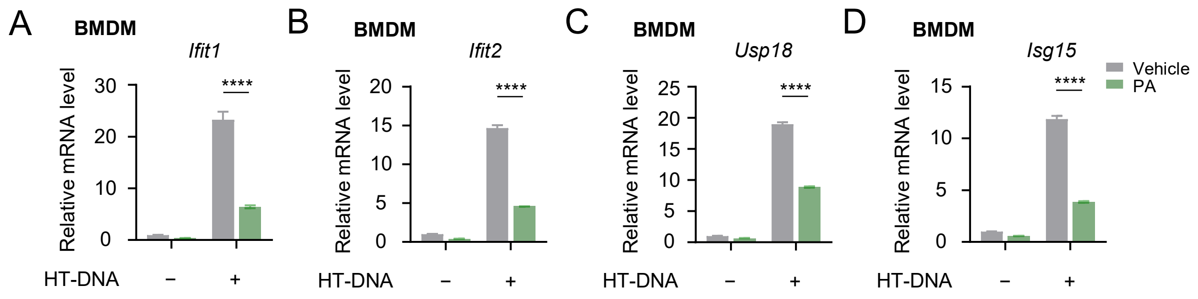

Supplementary Figure 2. Petroselinic acid suppresses cytosolic DNA-induced ISGs expression.

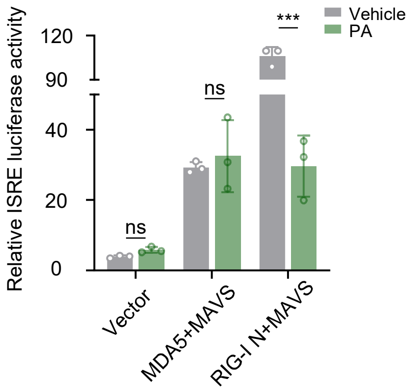

Supplementary Figure 3. Petroselinic acid fails to inhibit ISRE activation driven by MDA5/MAVS.

A

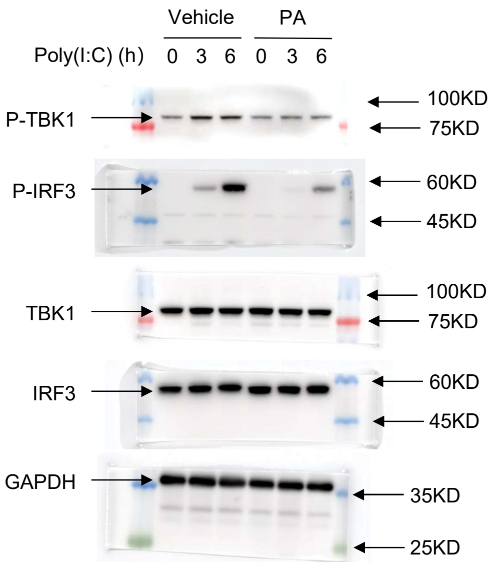

B

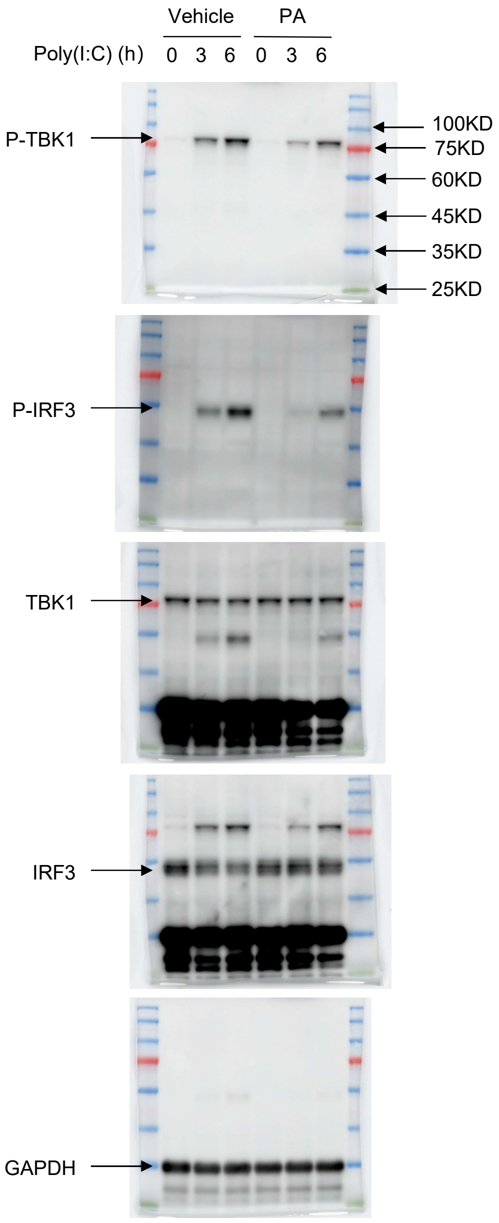

Supplementary Figure 4. Original Western blot images of Figure 4C,F.

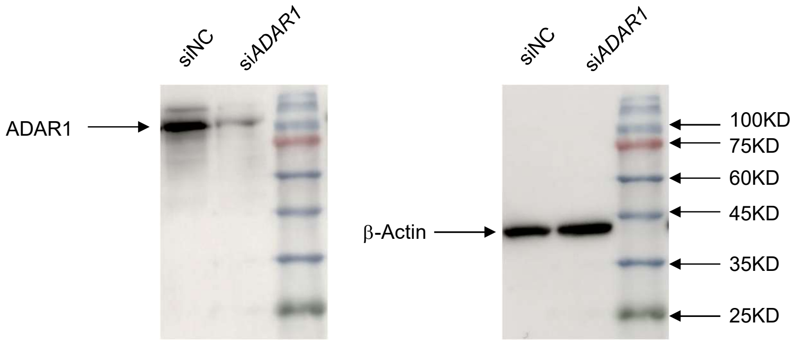

Supplementary Figure 5. Original Western blot images of Figure 6B.

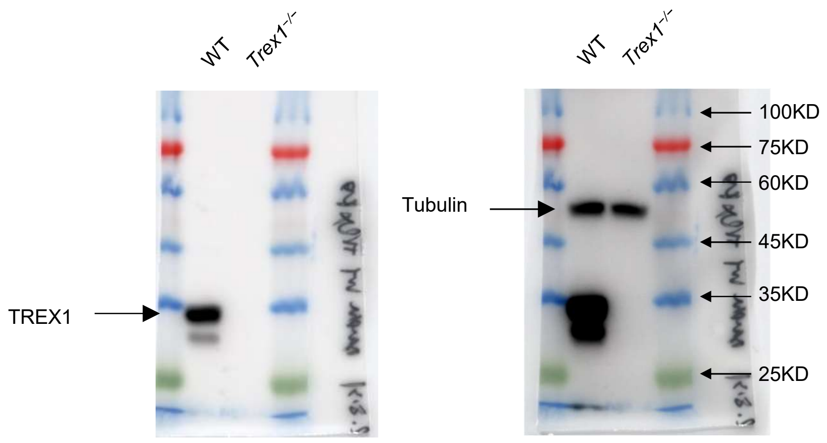

Supplementary Figure 6. Original Western blot images of Figure 7C.
