# Supplementary material for: Petroselinic Acid from Apiaceae Family Plants Ameliorates Autoimmune Disorders Through Suppressing Cytosolic-Nucleic-Acid-Mediated Type I Interferon Signaling
Source: Biomolecules. 2025 Feb 24;15(3):329. doi: 10.3390/biom15030329 (PMC11939978; doi:10.3390/biom15030329)
Supplement: Supplementary file 1 [file biomolecules-15-00329-s001.zip › 20250224 Supplementary information.pdf]

Supplementary figure legends

Supplementary Figure 1. Petroselinic acid suppresses cytosolic RNA-induced ISGs expression. (A-D) RT-qPCR analysis of *Ifit1* **(A)**, *Ifit2* **(B)**, *Usp18* **(C)** and *Isg15* **(D)** mRNA expression in BMDM cells transfected with 1μg/mL poly(I:C) following 200μM PA pretreatment for 6h. For A-D, P values represent comparison with vehicle calculated using log rank test. All values are mean ± s.d. ** $p \leqslant 0.01$; **** $p \leqslant 0.0001$; by unpaired t test.

Supplementary Figure 2. Petroselinic acid suppresses cytosolic DNA-induced ISGs expression. (A-D) RT-qPCR analysis of *Ifit1* **(A)**, *Ifit2* **(B)**, *Usp18* **(C)** and *Isg15* **(D)** mRNA expression in BMDM cells transfected with 1μg/mL HT-DNA following 200μM PA pretreatment for 6h. For A-D, P values represent comparison with vehicle calculated using log rank test. All values are mean ± s.d. **** $p \leqslant 0.0001$; by unpaired t test.

Supplementary Figure 3. Petroselinic acid fails to inhibit ISRE activation driven by MDA5/MAVS. Dual-luciferase reporter assays of ISRE reporter activation in HEK293T cells transfected with the indicated plasmids, followed by treatment with 200μM PA. P values represent comparison with vehicle calculated using log rank test. All values are mean ± s.d. n.s., not significant; *** $p \leqslant 0.001$; by unpaired t test.

Supplementary Figure 4. Original Western blot images of Figure 4C,F. (A) Original Western blot images of Figure 4C. **(B)** Original Western blot images of Figure 4F.

Supplementary Figure 5. Original Western blot images of Figure 6B.

Supplementary Figure 6. Original Western blot images of Figure 7C.

Table S1. Free binding energy and interacting amino acids for each inhibitor to cGAS

Inhibitors	Free binding energy (kcal/mol)	Interacting amino acids
PA	−52.9675	S435, Y436, K439
RU.521	−41.4829	S435, Y436, K439
G108	−53.2325	R376, Y436, L490
PF-06928215	−54.1303	K362, R376, E383, Y436

Table S2. Free binding energy and interacting amino acids for each inhibitor to RIG-I

Inhibitors	Free binding energy (kcal/mol)	Interacting amino acids
PA	−56.5257	P301, K849, K858
RIG012	−36.0515	R664, V699
Cyclo (Phe-Pro)	−20.3592	RIG-I 2CARD domain
